# A multi-site ^99m^Tc-HMPAO SPECT study of cerebral blood flow in a community sample of patients with major depression

**DOI:** 10.1038/s41398-024-02961-5

**Published:** 2024-06-03

**Authors:** Bradley S. Peterson, Jennifer Li, Manuel Trujillo, Siddhant Sawardekar, David Balyozian, Siddharth Bansal, Bernice F. Sun, Courtney Marcelino, Anoop Nanda, Tracy Xu, Daniel Amen, Ravi Bansal

**Affiliations:** 1 Institute for the Developing Mind, Children’s Hospital Los Angeles, Los Angeles, CA USA; 2 Department of Psychiatry, Keck School of Medicine at the University of Southern California, Los Angeles, CA USA; 3 Department of Psychiatry at NYU Grossman School of Medicine, New York, NY USA; 4 Amen Clinics Inc., Costa Mesa, CA USA

**Keywords:** Depression, Neuroscience

## Abstract

Prior regional Cerebral Blood Flow (rCBF) studies in Major Depressive Disorder (MDD) have been limited by small, highly selective, non-representative samples that have yielded variable and poorly replicated findings. The aim of this study was to compare rCBF measures in a large, more representative community sample of adults with MDD and healthy control participants. This is a cross-sectional, retrospective multi-site cohort study in which clinical data from 338 patients 18–65 years of age with a primary diagnosis of MDD were retrieved from a central database for 8 privately owned, private-pay outpatient psychiatric centers across the United States. Two ^99m^Tc-HMPAO SPECT brain scans, one at rest and one during performance of a continuous performance task, were acquired as a routine component of their initial clinical evaluation. In total, 103 healthy controls, 18–65 years old and recruited from the community were also assessed and scanned. Depressed patients had significantly higher rCBF in frontal, anterior cingulate, and association cortices, and in basal ganglia, thalamus, and cerebellum, after accounting for significantly higher overall CBF. Depression severity associated positively with rCBF in the basal ganglia, hippocampus, cerebellum, and posterior white matter. Elevated rCBF was especially prominent in women and older patients. Elevated rCBF likely represents pathogenic hypermetabolism in MDD, with its magnitude in direct proportion to depression severity. It is brain-wide, with disproportionate increases in cortical and subcortical attentional networks. Hypermetabolism may be a reasonable target for novel therapeutics in MDD.

## Introduction

Regional Cerebral Blood Flow(rCBF) is valuable in studies of normal and pathological brain processes because it is usually tightly coupled to metabolism [1–5], which facilitates physiological interpretation of findings compared to those of BOLD fMRI studies. Despite the value of rCBF measures, prior SPECT and PET studies of Major Depressive Disorder(MDD) have yielded limited insight into the pathogenesis of depression, primarily because findings have been highly variable across studies. This variability has been attributed to small sample sizes, differing sample characteristics, variable use of medication, and use of differing imaging modalities and different radiotracers [6, 7]. Sample sizes have typically been limited to 10–20 per group, and they have differed in their age and sex composition, which are known to affect rCBF. Studies also differ in their inclusion of co-occurring illnesses, with most studies allowing few or none, which has limited the generalizability of findings.

Another source of variability is whether and how studies adjusted or “normalized” for whole brain CBF (wbCBF). Normalization aims to improve quantification by controlling for random effects between participants, which can derive either from variations in radiotracer dosing or from individual differences in physiology that affect tracer levels. Normalization is helpful in small studies to reduce variance and improve statistical power, though large studies profit less in this regard [8]. Normalization can also alter findings and complicate their interpretation [8, 9]. If the patient group has a higher mean wbCBF, for example, normalizing for wbCBF can remove significant regional group differences, and it can yield brain regions that have reduced normalized but higher non-normalized rCBF values compared to controls. Early studies often normalized by dividing rCBF values region-wise or voxel-wise by rCBF measured in a pre-determined “reference” region, often the cerebellum or visual cortex, under the dubious assumption that these regions have normal rCBF values [8, 10]. Because a ratio multiplies noise in the numerator and denominator, more recent studies usually normalize rCBF using linear regression to covary for wbCBF.

We compared rCBF from ^99m^Tc-HMPAO SPECT scans acquired at rest and during a continuous performance task (CPT) in a community sample of 338 patients with MDD and 103 healthy controls. We assessed group differences in both normalized and non-normalized measures of rCBF, both at rest and during performance of a continuous performance task. We also assessed the moderation of group differences in rCBF by age and sex, and we assessed the associations of rCBF with depression severity. We hypothesized that normalized resting rCBF would differ significantly between groups and would associate significantly with depression severity. The great variability of prior findings in prior rCBF studies of depression precluded specifying a directionality to the hypothesized group differences.

## Materials and methods

### Study design

This is a retrospective, case-control study of deidentified clinical data collected at the Amen Clinics, a network of 8 privately owned, private-pay outpatient psychiatric centers across the United States. As a component of their initial clinical evaluation, patients routinely undergo ^99m^Tc-HMPAO SPECT scanning after a discussion of its risks and benefits. We retrieved clinical records and SPECT data from a central database for the clinical network.Patient Inclusion Criteria: (1) all consecutive patients who underwent initial evaluation between 2000 and 2013 at any one of the 8 clinics; (2) 18–65 years old at the time of evaluation; (3) received a primary clinical diagnosis of MDD that the evaluating clinician deemed to be primarily responsible for their clinic referral.Patient Exclusion Criteria: a lifetime diagnosis of dementia, bipolar illness, substance dependence, seizure disorder, or concussion. Record retrieval based on these criteria yielded a sample of 338 patients with a primary diagnosis of MDD. They were evaluated and scanned at 1 of 8 network clinics: Newport Beach=169; Virginia=55, Washington DC = 43, Seattle=28, Brisbane California=14, Fairfield California=20; Manhattan=5, Atlanta=4. SPECT scans and questionnaires were available from a control group of 103 healthy adults recruited through newspaper advertisements and flyers. They were all scanned at the Newport Beach clinic under identical procedures as patients.Control Inclusion Criteria: (1) age 18–65; (2) in good general health.Control Exclusion Criteria: (1) lifetime history of psychiatric diagnosis, concussion, or substance abuse; (2) use of psychotropic medication; (3) first-degree relatives with a psychiatric diagnosis.

### Institutional Review Board

Because this study analyzed deidentified data originally collected for clinical purposes, it was not considered human participant research by the Institutional Review Board of Children’s Hospital Los Angeles and was exempted from review. Healthy controls provided informed written consent under the Alpha IRB. All methods were carried out in accordance with relevant guidelines and regulations.

### Patient characterization

The initial clinical evaluation at each clinic was performed by a board-certified psychiatrist who assigned clinical diagnoses using DSM criteria and who identified one diagnosis to be the primary reason for clinical referral. Patients completed questionnaires for demographic, medical, medication, and social history, and a Beck Depression Inventory [11]. Sex, gender, race, and ethnicity were self-reported. They also completed symptom checklists probing DSM criteria for psychiatric diagnoses, from which we summed the endorsed symptoms to create a measure of symptom severity for each diagnosis (Supplemental Methods).

### SPECT scanning

Two SPECT scans were acquired for each participant. The first was obtained after performing the Conner’s Continuous Performance Test II (CPT-II) under normal lighting conditions. The radiopharmaceutical was injected 3 min after beginning the CPT, which lasted ~15 min; the scan was obtained 30 min after injection. At least 24 h later, the second SPECT scan was acquired 30 min after injection of radioligand while the participant rested with eyes open in a dimly lit room.

Brain SPECT data were acquired using identical scanners and procedures at all 8 sites. Scans were acquired using a Picker (Philips) Prism XP 3000 scanner equipped with a triple-headed gamma camera (Picker Int., Bedford Hills, OH) with low energy, high-resolution fan beam collimators. In each scan session, 120 images were acquired with a 128 × 128 matrix, each image separated by a 3^o^ angle, covering the entire head. SPECT data were attenuation-corrected using the general linear method [12]. Each participant was administered an intravenous dose of technetium-99m hexamethyl propylene amine oxime (^99m^Tc-HMPAO), standardized identically for age and weight across all sites.

### Image processing

We generated a histogram of voxel intensities for SPECT data across each brain that contained a higher-intensity peak representing voxels within the brain and a lower-intensity peak representing voxels outside the brain. The trough between the two peaks was selected as a threshold identifying non-brain voxels in the SPECT data. We then used a 3D region-growing algorithm to define a volume of connected voxels outside the brain by limiting region-growing to voxels with intensities smaller than the threshold. We used this 3D region to mask out all non-brain voxels, ensuring preservation of SPECT data within white matter. We used an affine transformation to register each participant’s masked SPECT image into the coordinate space of a SPECT template brain (Supplemental Methods) such that it maximized mutual information [13] between the template and masked participant image [14]. We then assessed visually the accuracy of spatial registration and adjusted registration parameters manually, if needed.

### Statistical analyses

We calculated wbCBF for each participant as the average SPECT data across all voxels in the brain. All statistical models were performed twice, once when covarying for wbCBF and once without covarying for it, to assess statistical effects on both normalized and non-normalized rCBF values. Age and sex were included as covariates in all analyses.

#### Hypothesis testing

A priori hypothesis testing was performed using rCBF at rest. Our primary analyses included only the 169 MDD patients scanned at the Newport site, because all 103 control participants were also scanned at that site; these analyses eliminated any undetected site effects and thereby provided the best controlled comparison of rCBF between depressed and control participants. Group differences were assessed using a general linear regression model: ${rCBF}={\beta }_{0}+{\beta }_{1}* {Group}+{\beta }_{2}* {Age}+{\beta }_{3}* {Sex}+{\beta }_{4}* {wbCBF}+\epsilon$.

We also assessed whether age or sex moderated group differences in resting rCBF by assessing the significance of group*age or group*sex interaction terms added separately to this regression model. We further assessed the association of resting rCBF in patients with depression severity as measured using the Beck Depression Inventory.

#### Secondary analyses

(1) We repeated all analyses for a priori hypothesis testing using data from the larger cohort of 338 depressed patients scanned across all 8 clinical sites, providing greater statistical power and improved generalizability of findings across a more broadly representative sample of the US population. The regression models in these analyses also covaried for site, using 7 dummy variables coded as 0 or 1, with Newport as the reference site. (2) We repeated all statistical analyses using rCBF measured during performance of the CPT to assess whether demands on attention and executive functioning affected our findings.

#### Sensitivity analyses

(1) To ensure that findings were attributable to the effects of depression and not a co-occurring disorder, we reran group comparison models while covarying for age and sex, but excluding patients with co-occurring anxiety disorder, ADHD, OCD, eating disorder, or substance use disorder. (2) To ensure that medication use was not affecting our findings, we repeated all analyses while excluding patients who were taking any psychotropic medication at the time of the SPECT scan. (3) We further assessed the effects of each medication on rCBF within the depressed group. In a single regression model with rCBF as the dependent variable, we included a term for each medication class (SSRI, SNRI, antipsychotic, anticonvulsant, or lithium), coded as either 0 or 1, while covarying for age and sex (with or without covarying for wbCBF).

#### Multiple comparisons

We corrected all *P* value maps for multiple comparisons using cluster false discovery rate (FDR) at a cluster defining threshold of u = 2.5 and FDR = 0.05 [15]. We color-coded and displayed FDR-corrected *P* values on the ICBM consortium T1 brain [16] to aid localization of findings.

## Results

### Sample characteristics

See Table 1.

**Table 1:** Participant demographics.

	Healthy adults	Depressed Newport adults	Test statistic (vs. healthy)	*df*	*P* value	Depressed adults all sites	Test statistic (vs. healthy)	*df*	*P* value
*n*	103	169				338			
Mean age, years, mean ± SD	39.4 ± 12.8	36.5 ± 13.7	*t* = 1.76	227.4	0.079	35.8 ± 13.6	t = 2.48	178.65	0.014
Age range, years	18–65	18–65				18–65			
Sex, % male	40.8%	46.2%	*χ*^2^ = 0.55	1	0.46	44.1%	*χ*^2^ = 0.23	1	0.63
Ethnicity									
Caucasian	63	107				233			
Asian	7	5				19			
Hispanic	7	11				14			
African American	1	2				6			
Unknown	25	44				66			
Comorbidities									
Any anxiety disorder		138 (81.7%)				260 (76.9%)			
Generalized anxiety disorder		87 (51.5%)				117 (34.6%)			
ADHD		38 (22.5%)				43 (12.7%)			
Bipolar		0				0			
OCD		14 (8.3%)				38 (10.4%)			
PTSD		0				0			
Current suicidal ideation		73 (43.2%)				103 (30.5%)			
Previous suicide attempt		10 (5.9%)				19 (5.6%)			
Eating disorder		5 (3.0%)				11 (3.3%)			
Personality disorder		2 (1.2%)				3 (1.0%)			
Substance abuse disorder*		4 (2.4%)				5 (1.5%)			
Alcohol-related disorder		3 (1.8%)				3 (1.0%)			
Cannabis-related disorder		1 (0.6%)				1 (0.3%)			
Other substance-related disorder		1 (0.6%%)				2 (0.6%)			
Medications									
Any psychotropic	0	69 (40.8%)				125 (37.0%)			
SSRI	0	30 (17.8%)				47 (13.9%)			
SNRI	1 (1.0%)	10 (5.9%)				19 (5.6%)			
Antipsychotics	0	4 (2.4%)				18 (5.3%)			
Stimulants	0	2 (1.2%)				8 (2.4%)			
Benzodiazepines	1 (1.0%)	16 (9.5%)				31 (9.2%)			
Anticonvulsants	0	5 (3.0%)				9 (2.7%)			
Anticonvulsant mood stabilizers	0	5 (3.0%)				10 (3.0%)			
Lithium	0	1 (0.6%)				3 (0.89%)			
Atypical antidepressants	0	17 (10.1%)				26 (7.7%)			

Newport:

• Resting scans were not acquired in 22 patients with depression and 26 healthy controls.

• CPT scans were not acquired in 2 patients with depression and 1 healthy control.

• 1 healthy control was missing demographic information.

All sites:

• Resting scans were not acquired in 36 patients with depression and 26 healthy controls.

• CPT scans were not acquired in 2 patients with depression and 1 healthy control.

• 1 healthy control was missing demographic information.

^*^1 patient had both alcohol and cannabis-related disorder.

### Group differences: resting rCBF

#### Newport only

**Fig. 1:**
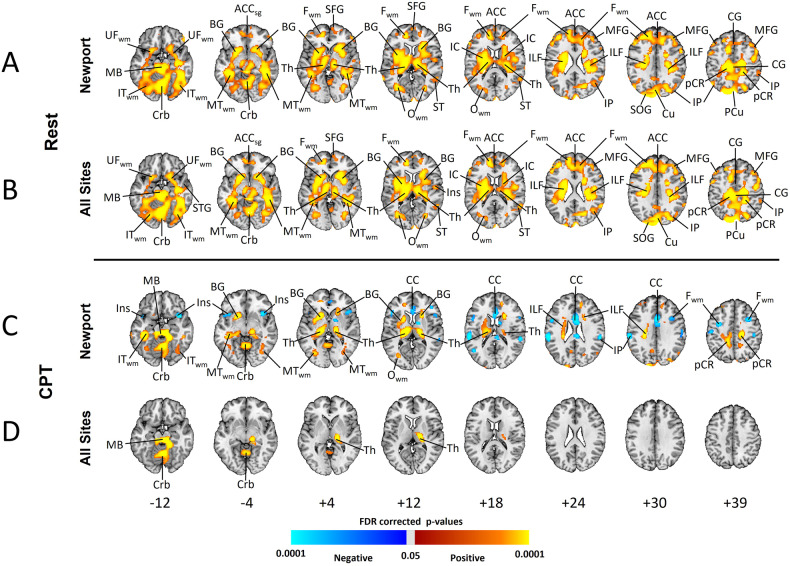
Group Comparisons in rCBF at Rest and During the CPT (Covarying for wbCBF). **A** and **B** are maps at rest. **C** and **D** are during performance of the CPT. All analyses control for the effects of age and sex, and analyses for all sites also control for site. *P* values that survived FDR correction at an FDR-corrected *P* < 0.05 were color-coded as shown in the color bar and displayed on the template brain, with warm colors representing higher rCBF and cooler colors representing lower rCBF values relative to wbCBF in the depressed compared with healthy control participants. The numbers below each column are axial slice level (in millimeters) in the Talairach coordinate system. The right sides of the images correspond to the right side of the brain. *Resting scans:* Newport Only Depressed: *N* = 147 (66 males, 81 females, mean age: 36.1 years); All 8 Sites Depressed: *N* = 302 (133 males, 169 females, mean age 35.5 years); Newport Only Healthy Controls: *N* = 78 (33 males, 45 females, mean age 37.9 years). *CPT scans:* Newport Only Depressed: *N* = 167 (76 males, 91 females, mean age: 36.6 years); All 8 Sites Depressed: *N* = 336 (147 males, 189 females, mean age 35.7 years); Newport Only Healthy Controls: *N* = 103 (43 males, 60 females, mean age: 39.6 years). ACC anterior cingulate cortex, ACC_sg_ subgenual portion of anterior cingulate cortex, BG basal ganglia, CC corpus callosum, CG cingulate gyrus, Crb cerebellum, F_WM_ frontal lobe white matter, Hi hippocampus, IC internal capsule, IFG inferior frontal gyrus, IL inferior longitudinal fasciculus, Ins insula, IP inferior parietal, IT_WM_ Inferior temporal white matter, MB midbrain, MFG middle frontal gyrus, MT_WM_ middle temporal white matter, O_WM_ occipital white matter, pCR posterior corona radiata white matter, PCu precuneus, SFG superior frontal gyrus, SOG superior occipital gyrus, STG superior temporal gyrus, Th thalamus, UF_WM_ uncinate fasciculus white matter.

rCBF was elevated relative to wbCBF in the anterior cingulate cortex (ACC), basal ganglia (BG), thalamus, midbrain, the superior (SFG) and middle frontal (MFG) gyri, inferior parietal lobe (IPL), precuneus, cuneus, and cerebellum, and white matter of the frontal (F_WM_), temporal (T_WM_), and occipital (O_WM_) lobes, internal capsule (IC), inferior longitudinal fasciculus (ILF), and uncinate fasciculus (UF_WM_) (Fig. 1A and Supplementary Fig. 1A).

#### All sites

Findings were similar to those for Newport participants (Fig. 1B and Supplementary Fig. 1B).

#### Both analyses

When not covarying for wbCBF, rCBF was higher in MDD participants throughout the entire brain (Supplementary Fig. 1E, rows A and B). Findings were similar when excluding patients taking psychotropic medications (Supplementary Fig. 1F) and when excluding patients with comorbid disorders (Supplementary Fig. 1G).

### Group differences: during CPT

#### Newport only

rCBF was elevated relative to wbCBF in the BG, thalamus, cerebellum, ILF, and T_WM_, and lower in the insula, IPL, F_WM_, and CC (Fig. 1C and Supplementary Fig. 1C).

#### All sites

rCBF was elevated relative to wbCBF in the thalamus, midbrain, and cerebellum (Fig. 1D and Supplementary Fig. 1D).

#### Both analyses

rCBF was globally increased in MDD participants when not covarying for wbCBF (Supplementary Fig. 1E, rows C and D).

### Moderation by age

#### Resting rCBF, Newport only

**Fig. 2:**
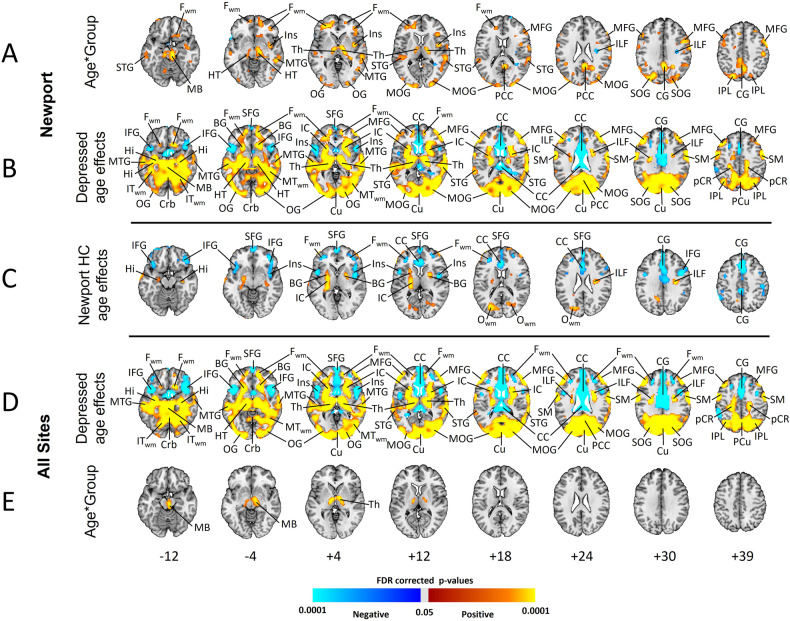
Age Moderation of Group Differences in Resting rCBF (Covarying for wbCBF). **A** tests the age*group interaction in Newport-only participants, and **E** tests the interaction in depressed patients across all 8 sites (and healthy controls scanned at the Newport site). **B** and **D** show significant age effects in depressed patients from Newport or all sites, respectively, and **C** shows age effects in healthy controls, with warm colors representing increasing rCBF with age relative to wbCBF, and cool colors representing increases in rCBF with age that are less than the increases in wbCBF with age (see Supplementary Fig. 2A). All analyses included the main effects of age and sex, and they covaried for wbCBF. Analyses for all sites also controlled for site with seven dummy variables. Voxels with an FDR-corrected *P* < 0.05 were color-coded as shown in the color bar. Number of participants and abbreviations are as in Fig. 1.

The age*group interaction was significant in F_WM_, T_WM,_ MFG, middle (MTG) and superior (STG) temporal gyri, insula, thalamus, midbrain, hypothalamus, IPL, occipital gyri, posterior cingulate cortex (PCC), and cingulate gyrus (CG) (Fig. 2A), deriving from a steeper decline in rCBF with age in MDD patients than controls in IFG, MFG, and insula, and increasing rCBF with age in MDD patients that was absent in controls in temporal gyri, thalamus, midbrain, IPL, STG, MTG, and occipital gyri (Fig. 2B, C). Findings were similar when not covarying for wbCBF but were spatially much more expansive (Supplementary Fig. 2A, row A), and no age-related decline in cortical regions was present in MDD participants (Supplementary Fig. 2A, rows B and C). Scatterplots showed these interactions were not driven by outliers (Supplementary Fig. 2A, bottom).

#### Resting rCBF, all sites

A significant age*group interaction was detected only in the midbrain and thalamus when covarying for wbCBF (Fig. 2E). When not covarying for wbCBF, findings were similar to Newport-only participants (Supplementary Fig. 2A).

#### During CPT, Newport only

When covarying for wbCBF, age*group interactions were sparse (Supplementary Fig. 2B, row A) and derived from a greater increase in rCBF with age in the midbrain, thalamus, and occipital gyri, and a greater age-related decline in the IFG, in depressed patients compared to controls (Supplementary Fig. 2B, rows B and C). When not covarying for wbCBF, the age*group interaction was significant in most of the brain (Supplementary Fig. 2C, row A), deriving from declining rCBF with age in SFG, MFG, IFG, T_WM_, IPL, and CG in controls but not MDD patients, and increasing rCBF with age in much of the remaining brain in MDD patients but not controls (Supplementary Fig. 2C, rows B and C).

#### During CPT, all sites

No significant interaction was detected when including patients from all sites and covarying for wbCBF (Supplementary Fig. 2B, row E). When not covarying for wbCBF, the age*group interaction was significant throughout the brain (Supplementary Fig. 2C, row E), mirroring Newport-only findings.

### Moderation by sex

#### Resting rCBF, Newport only

**Fig. 3:**
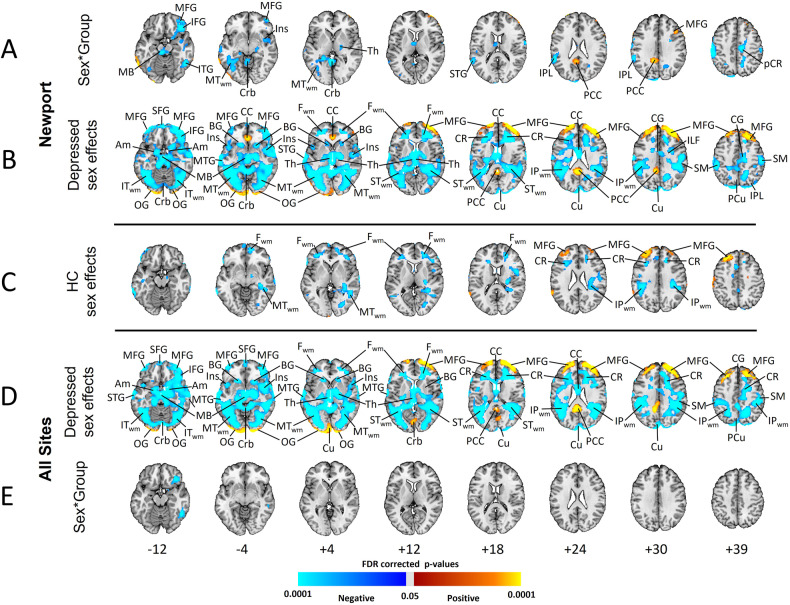
Sex Moderation of Group Differences in Resting rCBF (Covarying for wbCBF). **A** tests the sex*group interaction in Newport-only participants, and **E** tests the interaction in depressed patients across all 8 sites (and healthy controls scanned at the Newport site). **B** and **D** show significant sex effects in depressed patients from Newport or all sites, respectively, and **C** shows sex effects in healthy controls, with warm colors representing higher rCBF in males and cool colors representing higher rCBF in females (see Supplementary Fig. 3A). All analyses included the main effects of age and sex, and they covaried for wbCBF. Analyses for all sites also controlled for site with seven dummy variables. Voxels with an FDR-corrected *P* < 0.05 were color-coded as shown in the color bar. Number of participants and abbreviations are as in Fig. 1.

When covarying for wbCBF, the sex*group interaction was significant in MFG, IFG, CG, T_WM_, midbrain, cerebellum, thalamus, and IPL (Fig. 3A). When not covarying for wbCBF, this interaction was significant throughout the brain (Supplementary Fig. 3A). The interaction derived from greater rCBF in female MDD participants (Fig. 3B, C and Supplementary Fig. 3A, rows B and C).

#### During CPT

Findings were similar to rest (Supplementary Fig. 3B, C).

### Associations with symptom severity

**Fig. 4:**
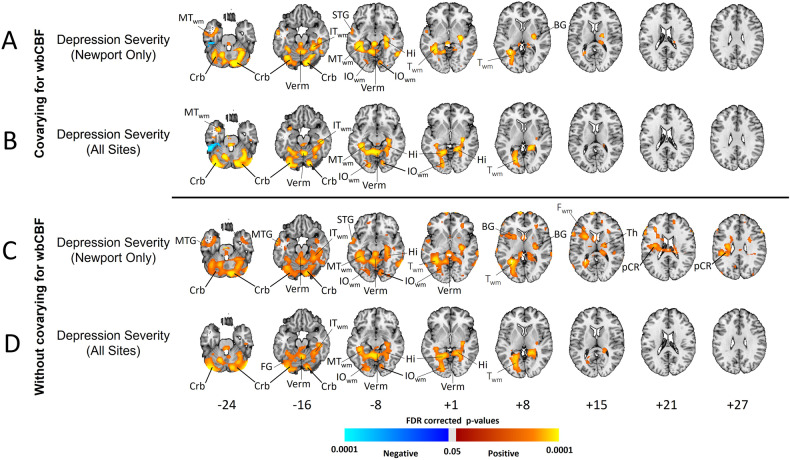
Associations of Resting rCBF with Depression Symptom Severity. These analyses that assessed the association of depression symptom severity on the Beck Depression Inventory (BDI) with resting rCBF were performed in the depressed patients only while covarying for age and sex. Models generating the maps in (**A**, **B**) also covaried for wbCBF, whereas models generating maps in (**C**, **D**) did not. Analyses for depressed patients at all sites (**B**, **D**) also controlled for site. Voxels with an FDR-corrected *P* < 0.05 were color-coded as shown in the color bar. Newport-Only Patients *N* = 122 (49 males, 73 females, mean age 37.0 years); All Sites Patients *N* = 241 (96 males, 145 females, mean age 36.2 years). These analyses had fewer depressed patients than Fig. 1 because some were missing BDI scores. Abbreviations are as in Fig. 1.

Depression symptom severity associated positively with rCBF in the hippocampus, cerebellum, BG, and T_WM_ for Newport-only participants and all sites, and when covarying for wbCBF or not (Fig. 4A–D). Findings persisted when covarying for anxiety symptom severity and wbCBF (Supplementary Fig. 4A, rows A and B) and included positive associations in the BG and subgenual ACC. When covarying for anxiety symptom severity but not wbCBF, this positive correlation was present throughout the brain (Supplementary Fig. 4B, rows A and B and Supplementary Fig. 4C).

### Medication effects

SSRI use (*N* = 47) was associated with lower perfusion in cortical and subcortical gray matter; antipsychotic use (*N* = 18) was associated with global hyperperfusion (Supplementary Fig. 5).

### Task-related change in rCBF

To assess the dynamic change in rCBF during concentration, we subtracted rCBF at rest from rCBF when performing the CPT voxel-wise, but it yielded no significant findings for any model.

## Discussion

### Resting rCBF

#### Group differences

When covarying for wbCBF, depressed patients had elevated rCBF in frontal, anterior cingulate (ACC), parietal (PC), superior temporal (ST), and posteroinferior temporal (IT) cortices, and in the basal ganglia (BG), thalamus, and cerebellum (Fig. 1). When not covarying for wbCBF, rCBF was elevated everywhere in the brain (Supplementary Fig. 1E). Thus, findings when controlling for wbCBF were in regions where rCBF was disproportionately elevated above the overall increase in blood flow. Findings were unchanged when excluding patients taking psychotropic medications (Supplementary Fig. 1F).

#### Associations with symptom severity

Depression severity associated positively with rCBF in the BG, hippocampus, cerebellum, and posterior white matter, with or without covarying for wbCBF (Fig. 4). When covarying for anxiety severity but not wbCBF, depression severity associated positively with rCBF throughout the brain (Supplementary Fig. 4B, rows A and B).

#### Interpretation

Frontal, ACC, BG, thalamus, hippocampus, and cerebellar regions constitute interconnected networks that subserve attention, reward, and regulatory processes [17, 18]. Abundant clinical and preclinical evidence has implicated these networks in the pathophysiology of depression [19], and their abnormal metabolism could contribute to the problems with concentration, attention, other cognitive processes, and mood dysregulation long observed in MDD [20].

Hypermetabolism in both gray and white matter suggests the presence of dysfunctional energetics in either neurons or glia or both. Elevated rCBF throughout the brain when not covarying for wbCBF suggests that the dysfunctional energetics in neurons or glia is global. Gray matter in cortex, basal ganglia, thalamus, and hippocampus is composed primarily of neuronal cell bodies and neurites, but they also contain in smaller proportions axons, myelin, and glia [21, 22], and therefore a metabolic disturbance affecting any of these cellular components could produce white and gray matter hyperperfusion [23, 24]. Abnormal “resting state” rCBF suggests the presence of disordered cellular housekeeping functions that maintain resting neuronal membrane potentials [25, 26], in which case elevated rCBF could represent an attempt at neuronal compensation. Significant positive associations with depression severity, however, suggest that hypermetabolism is more likely pathogenic than compensatory, since successful compensation would produce an inverse association of rCBF with symptom severity.

#### Moderators of group differences

Group differences depended significantly on age and sex of participants. rCBF increased significantly with age in frontal white matter, and in cingulate, ST, occipital, and insular cortices, and in the thalamus in depressed patients but not controls (Fig. 2 and Supplementary Fig. 2). Thus, older participants contributed disproportionately to elevated rCBF in these regions within the depressed group (Supplementary Fig. 2A bottom). Prior studies have reported that rCBF in healthy persons declines steadily with age throughout adulthood in most cortical regions [1–5, 27–32], but most prominently in the superior frontal gyrus and ACC [31–34], as we found in our controls. This normative age-related decline in rCBF is independent of cortical thinning [30] and parallels a decline in cerebral metabolic rate for oxygen (CMRO_2_), suggesting that declining rCBF in healthy persons derives from an age-related decline in neural activity or synaptic density [5], particularly in the ACC and inferior frontal gyrus. One prior study assessed the moderating effects of age on rCBF in MDD, with no significant effects detected [35]. The modifying effect of age on rCBF that we detected suggests that the pathology driving hyperperfusion in depression exacerbates with increased age, and that resting neuronal activity or synaptic density may increase with age in MDD. Because ours is a cross-sectional study, however, we cannot determine whether the age-related increase in rCBF would be observed in depressed persons followed over time or whether the finding represents a differing biological subtype in persons who present clinically with depression in later life.

rCBF was also significantly higher in depressed women (Fig. 3 and Supplementary Fig. 3). Prior studies have reported higher rCBF in healthy women than men in most brain regions [5, 36–43]; examination of figures in those studies suggest that elevations are greatest in white matter [42, 44], consistent with our findings in healthy controls (Supplementary Fig. 3). Some speculate that higher rCBF in women may derive from the vasodilating effects of estrogen in response to CO_2_ [45]. Whatever the causes, the neurobiological processes underlying elevated rCBF in women may be exaggerated in depressed women and could contribute to their 2-fold greater population prevalence of depression [46].

### rCBF during the CPT

Group differences lessened substantially during performance of the CPT (Fig. 1), perhaps because resting rCBF in depressed patients was near ceiling and had little capacity to increase further, whereas controls could increase flow to meet attentional task demands and thereby more closely approximate values in depressed patients. Higher rCBF was in similar regions as at rest (BG, thalamus, and brainstem), but less significant and much smaller in spatial extent; rCBF was lower relative to overall higher wbCBF in the insula and CC (Fig. 1). The moderating effects of age and sex (their interactions with group) during the CPT were statistically weaker but in similar locations as during resting scans (Supplementary Figs. 2B, C and 3B, C).

### Task-related change in rCBF

rCBF changes from rest to activation during the CPT did not differ significantly between groups and were not associated with depression symptom severity anywhere. Together with our overall similar findings for rCBF at rest and during the CPT, these findings suggest that metabolic disturbances in depression are trait-related, unassociated with changes in dynamic state.

### Medication effects

SSRI use was associated with lower rCBF throughout cortical and subcortical gray matter, whereas antipsychotic use was associated with globally higher rCBF in gray and white matter (Supplementary Fig. 5). Lower rCBF with SSRI use is consistent with conclusions of prior studies that antidepressant medication is associated with either a reduction or normalization of rCBF in MDD [47–49]. It is also consistent with our interpretation that hyperperfusion is pathogenic in MDD, and it suggests that the therapeutic effects of SSRIs may be attributable in part to their lowering gray matter metabolism. Group differences were unchanged when including medication use, age, sex, and the age-by-group and sex-by-group interactions as covariates in a single statistical model.

### Relation to previous studies

Consistent with our findings, elevated rCBF has been reported previously in depressed compared with healthy adults in: the ACC, BG, hippocampus, and cerebellum [50]; frontal cortex, BG, and thalamus [51]; insula [52]; cerebellum [53]; frontotemporal cortices [54]; BG, ACC, and amygdala [55]; white matter [56]; and prefrontal cortex and amygdala [57]. A voxel-wise meta-analysis of 12 studies of first-episode, medication-naïve depressed patients (*N* = 313) compared with healthy controls (*N* = 283) found elevated rCBF in amygdala, hippocampus, parahippocampus, supplementary motor area, and middle frontal gyrus, and reduced rCBF in lingual gyrus, middle occipital gyrus, cuneus, and cerebellum [58]. These prior findings and ours, however, stand in contrast to the conclusions of a systematic review and meta-analysis of 15 CBF studies that overall rCBF in depressed patients (*N* = 538) was slightly lower than in healthy participants (*N* = 416) (−2.24 ml/min/100 g, 95% CI −4.12, −0.36; *P* = 0.02), with moderate heterogeneity (*I*^2^ = 64%) [7], although these findings did not survive when excluding late life depression (age >60). Reductions in normalized rCBF were noted most commonly in the ACC, BG, thalamus, prefrontal and superior temporal cortices, and brainstem [7].

We suspect that normalizing regional rCBF by elevated mean wbCBF or reference ROI values may have contributed to the prior reports of reduced rCBF in frontal cortices and ACC. Our findings suggest that these regional reductions are only relative to elevated wbCBF. Further, our age-specific findings (Fig. 2 and Supplementary Fig. 2) suggest that these relative reductions are more prominent in middle-aged and older adults and may account for prior meta-analytic findings that the reductions were greatest in late-life depression.

### Limitations

Our sample of depressed adults was evaluated and treated in a network of private pay psychiatric clinics, and it likely excluded underserved patients. Nevertheless, the sample was drawn from 8 clinics across the nation, providing excellent geographic diversity and a sample size that is an order of magnitude larger than prior rCBF studies of MDD. Research-grade diagnostic evaluations and assessments of symptom severity were not obtained, though clinical diagnoses were made by board-certified psychiatrists during a comprehensive clinical evaluation.

## Conclusions

Hypermetabolism is likely pathogenic in depression, with its magnitude in direct proportion to the severity of depressive symptoms. It is brain-wide, with disproportionate increases in cortical and subcortical attentional networks, particularly in women and older patients. Future CBF studies should be sufficiently large to permit assessment of regional flow without wbCBF normalization, and they should include participants with a full range of clinical presentations to ensure findings generalize to real-world patients. SSRIs are associated with lower rCBF, which may account in part for their therapeutic effects. Hypermetabolism may be a reasonable target for novel therapeutics in MDD.

### Supplementary information

Supplemental Materials

## Data Availability

The data that support the findings of this study are available from the corresponding author, BSP, upon reasonable request.
