# Supplementary material for: A multi-site 99mTc-HMPAO SPECT study of cerebral blood flow in a community sample of patients with major depression
Source: Transl Psychiatry. 2024 Jun 3;14:234. doi: 10.1038/s41398-024-02961-5 (PMC11148018; doi:10.1038/s41398-024-02961-5)
Supplement: Supplementary file 1 — Supplemental Materials [file 41398_2024_2961_MOESM1_ESM.pdf]

Supplemental Materials
Table of Contents

Page #

<u>SECTION 1: SUPPLEMENTAL METHODS</u>.. 2
Selection of a SPECT Template Brain 2
Measure of Anxiety Symptom Severity 2

<u>SECTION 2: SUPPLEMENTAL FIGURES</u>...3
Group Differences... 4
SFig.1A: Group differences at rest – Newport-only patients, all slices................................ 5
SFig.1B: Group differences at rest – all sites, all slices...6
SFig.1C: Group differences during the CPT – Newport-only patients, all slices..................... 7
SFig.1D: Group differences during the CPT – all sites, all slices...8
SFig.1E: Group differences without covarying for wbCBF 9
SFig.1F: Group differences in medication-free participants 10
SFig.1G: Group differences when excluding patients with comorbid disorders..................11
Modifying Effects of Age on Group Differences.. 12
SFig.2A Modifying effects of age on resting rCBF without covarying for wbCBF.................. 13
SFig.2B Modifying effects of age on rCBF during CPT while covarying for wbCBF................14
SFig.2C Modifying effects of age on rCBF during CPT without covarying for wbCBF............15
Modifying Effects of Sex on Group Differences... 16
SFig.3A Modifying effects of sex on resting rCBF without covarying for wbCBF...................17
SFig.3B Modifying effects of sex on rCBF during CPT while covarying for wbCBF 18
SFig.3C Modifying effects of sex on rCBF during CPT without covarying for wbCBF 19
SFig.3D Bar graphs for the modifying effects of sex on group differences............................ 20
Associations of rCBF with Depression and Anxiety Symptom Severity...21
SFig.4A Associations with resting rCBF while covarying for wbCBF 22
SFig.4B Associations with resting rCBF without covarying for wbCBF 23
SFig.4C Scatterplots for depression severity associations.. 24
Medication Effects.. 25
SFig.5 Psychotropic medication effects on resting rCBF.. 26

SUPPLEMENTAL METHODS

Selection of a SPECT Template Brain We used a two-step procedure to identify a healthy participant brain as the SPECT template. First, we identified the brain of the demographically most representative healthy participant as a preliminary template and then affine-registered all healthy SPECT data to it. Then we identified the healthy participant whose registration parameters were closest to the average of the 3 translations, 3 rotations, and 3 scaling parameters across all healthy participants. This participant's brain was then selected as the final template

Measure of Anxiety Symptom Severity Patients completed intake questionnaires that included either "General Symptom Checklist" (GSC) consisting of questions about symptoms taken from DSM criteria for specific disorders, including depression, anxiety disorders, ADHD, obsessive compulsive disorder, Tourette syndrome, bipolar I disorder, post-traumatic stress disorder, anorexia, bulimia, and psychosis. Patients rated the severity of each symptom on a scale of 0 to 4 (0=never, 1=rarely, 2=occasionally, 3=frequently, or 4=very frequently). We created an anxiety symptom score that summed scores for GAD, panic disorder, social phobia, and simple phobia. The possible range of scores was from 0 to 52: "Please rate yourself on each of the symptoms listed below using the following scale."

<u>GAD</u>
- Being markedly more irritable or experiencing anger outbursts
- Having unrealistic or excessive worry in at least a couple areas of your life
- Trembling, twitching, or feeling shaky
- Experiencing muscle tension, aches, or soreness
- Having feelings of restlessness
- Becoming easily fatigued

<u>Panic Disorder</u>
- Experiencing shortness of breath or feeling smothered
- Experiencing a pounding or racing heartbeat
- Sweating or having cold, clammy hands
- Experiencing dizziness or lightheadedness
- Having nausea, diarrhea or other abdominal distress

<u>Social Phobia</u>
- Excessive fear of being judged by others, which causes you to avoid or get anxious in situations

<u>Simple Phobia</u>
- Experiencing persistent, excessive fears of heights, closed spaces, specific animals, etc.

SUPPLEMENTAL FIGURES

GROUP DIFFERENCES

Group Differences in Resting rCBF, Newport-Only Patients and Controls, All Slices

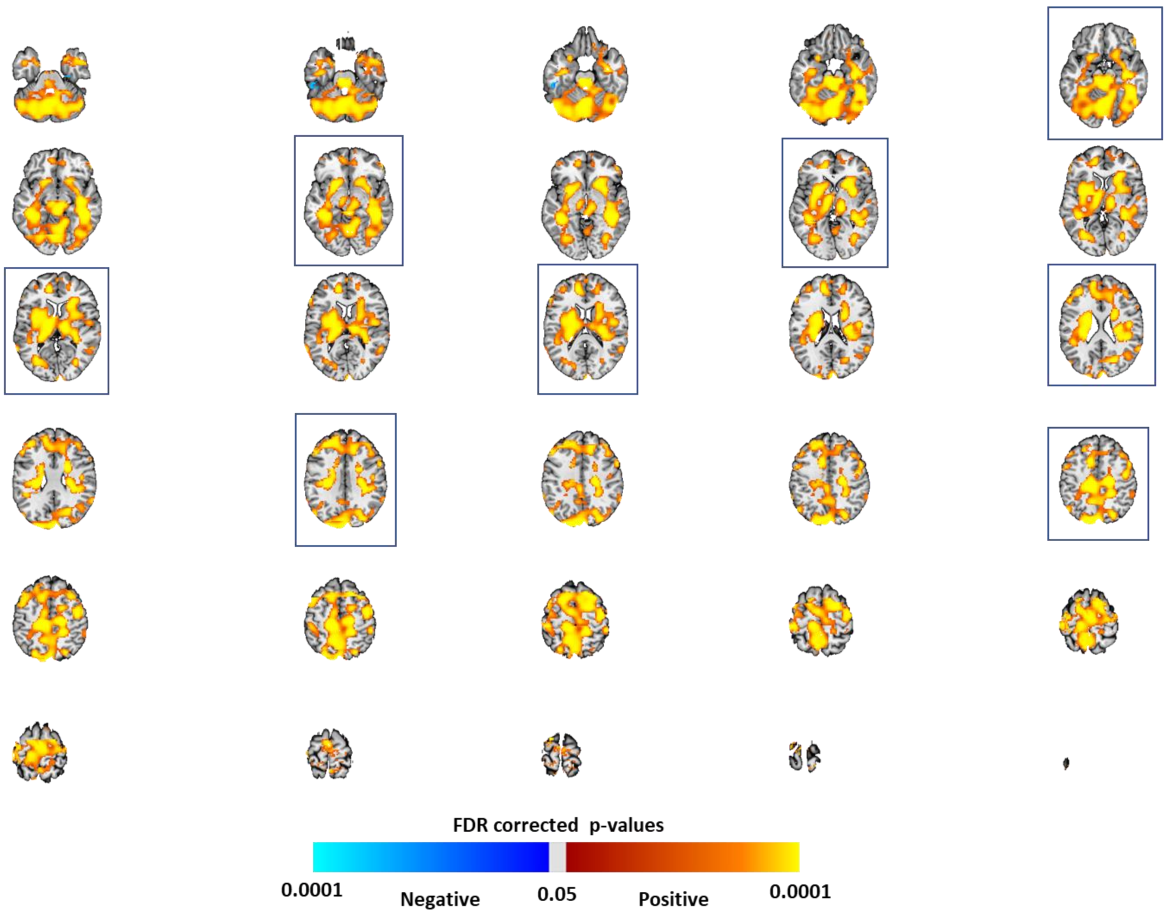

SFigure 1A. Group comparisons at rest while covarying for wbCBF – Newport-only patients, all slices
Covariates included age, sex, and wbCBF. Boxes indicate the slices shown in Figure 1 of the main text.

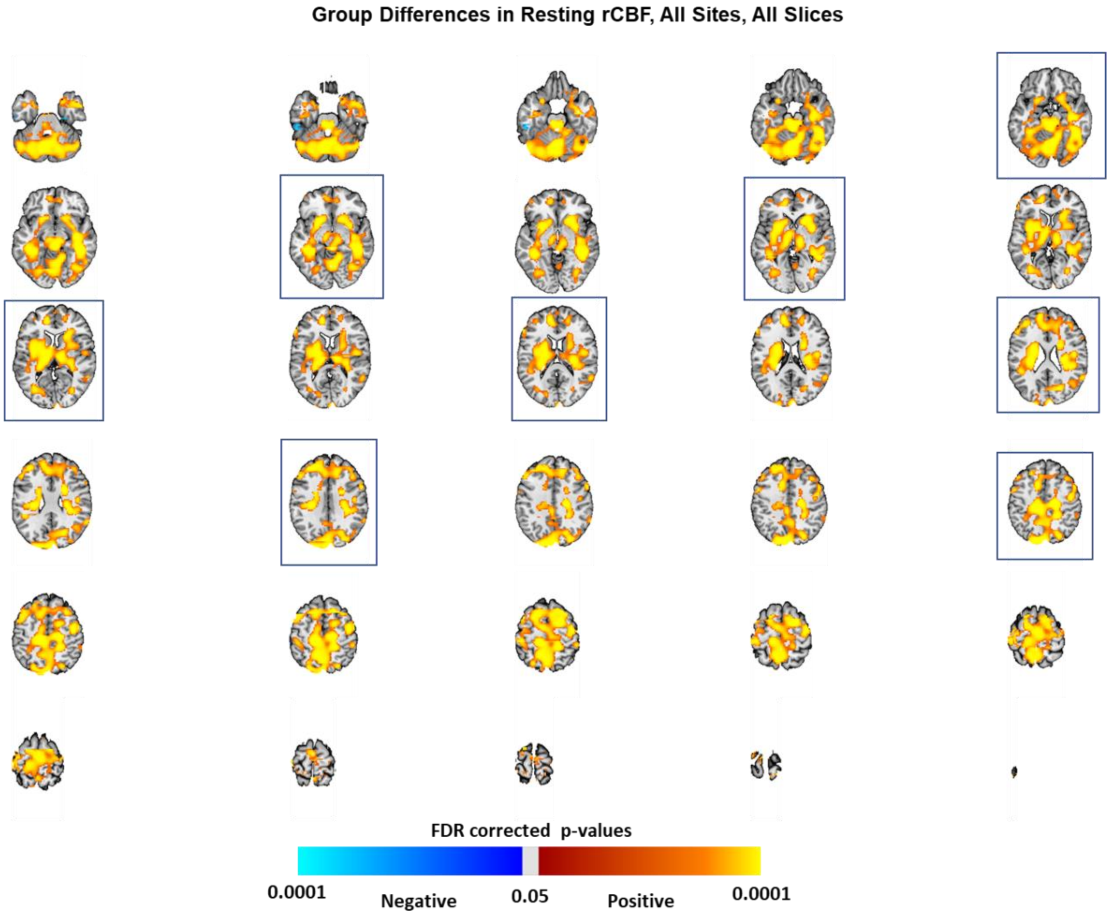

SFigure 1B. Group comparisons at rest while covarying for wbCBF – All sites, all slices
Covariates included age, sex, wbCBF, and site. Boxes indicate the slices shown in Figure 1 of the main text.

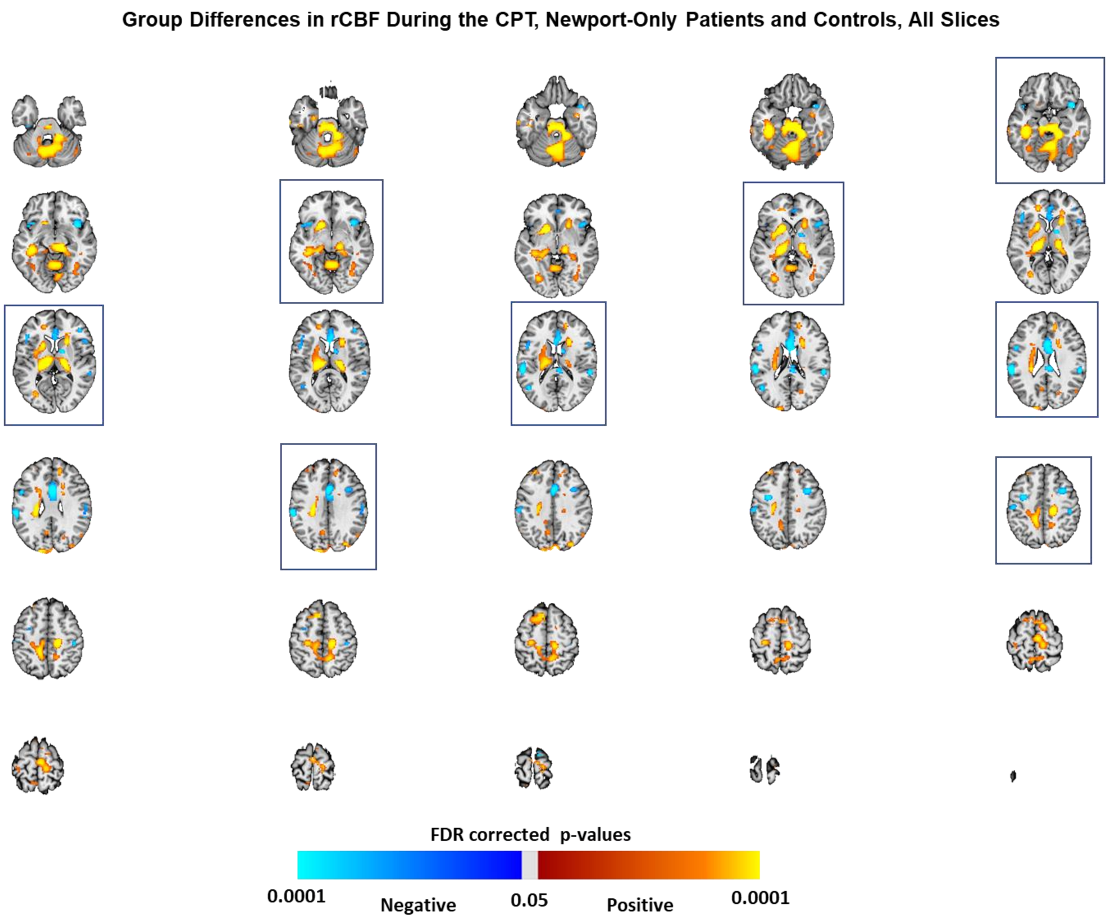

SFigure 1C. Group comparisons during the CPT while covarying for wbCBF – Newport-only patients, all slices

Covariates included age, sex, and wbCBF. Boxes indicate the slices shown in Figure 1 of the main text.

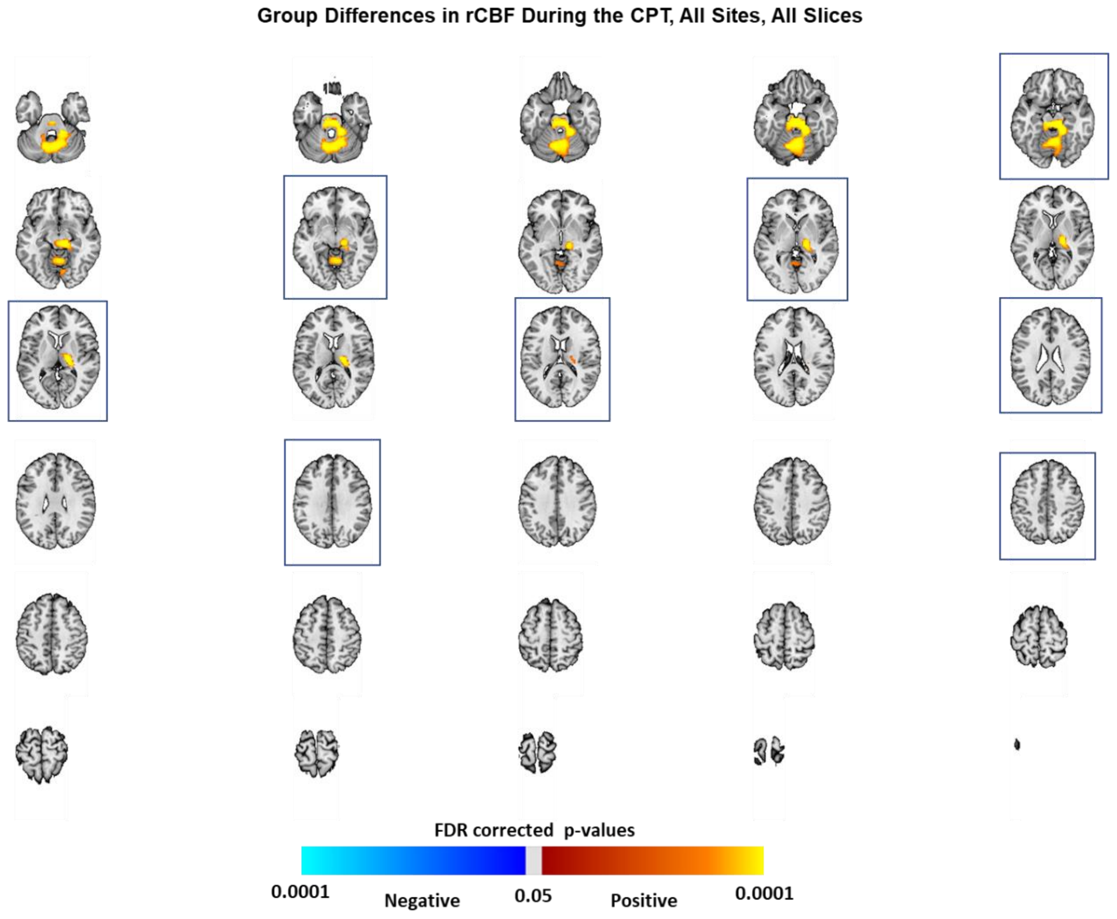

SFigure 1D. Group comparisons during the CPT while covarying for wbCBF – All sites, all slices
Covariates included age, sex, wbCBF, and site. Boxes indicate the slices shown in Figure 1 of the main text.

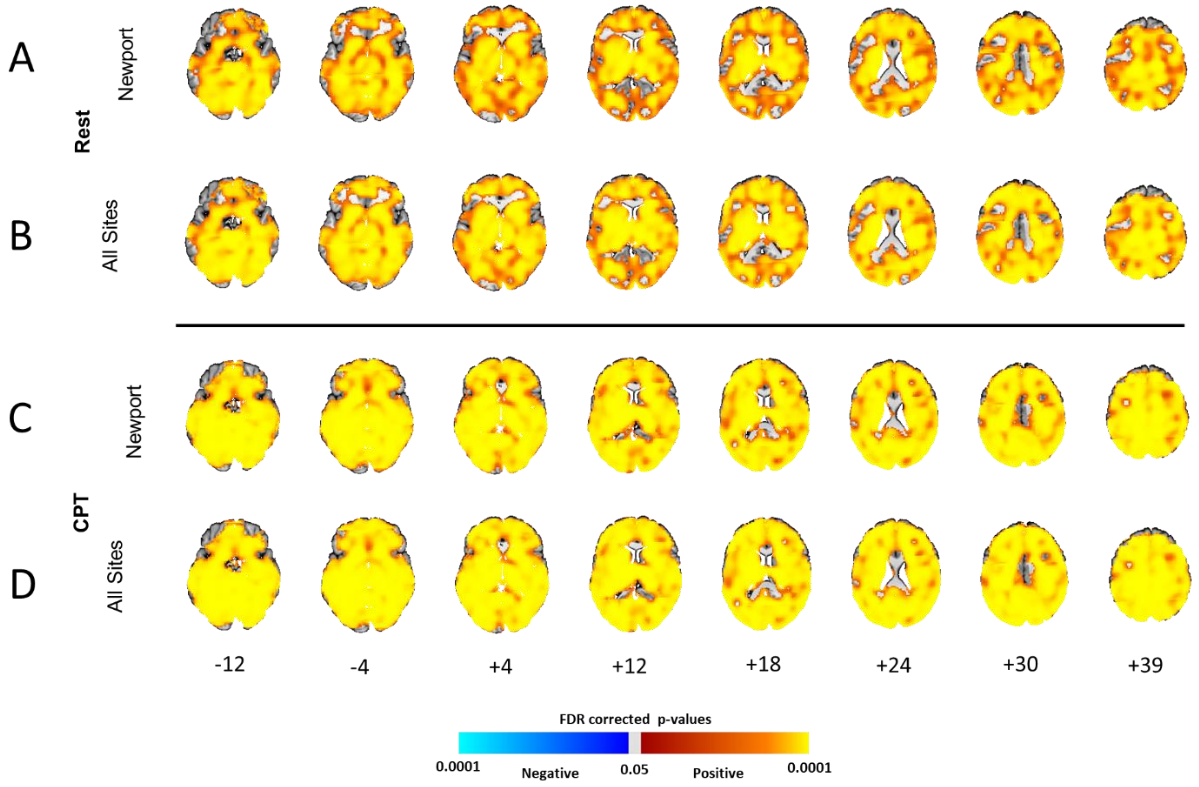

SFigure 1E. Group comparisons at rest and during CPT without covarying for wbCBF.

Rows A & B are maps at rest. Rows C & D are during performance of the CPT. All analyses control for the effects of age and sex, and analyses for all sites also control for site. P-values that survived FDR correction at an FDR-corrected $p < 0.05$ were color-coded as shown in the color bar and displayed on the template brain, with warm colors representing higher rCBF and cooler colors representing lower rCBF values in the depressed compared with healthy control participants. The numbers below each column are axial slice level (in millimeters) in the Talairach coordinate system. The right sides of the images correspond to the right side of the brain.

Resting Scans:

Newport Only Depressed: N=147 (66 males, 81 females, mean age: 36.1 years)
All 8 Sites Depressed: N=302 (133 Males, 169 Females, Mean Age 35.5 years)
Newport Only Healthy Controls: N=78 (33 males, 45 females, mean age 37.9 years)

CPT Scans:

Newport Only Depressed: N=167 (76 males, 91 females, mean age: 36.6 years)
All 8 Sites Depressed: N=336 (147 Males, 189 Females, Mean Age 35.7 years)
Newport Only Healthy Controls: N=103 (43 males, 60 females, mean age: 39.6 years)

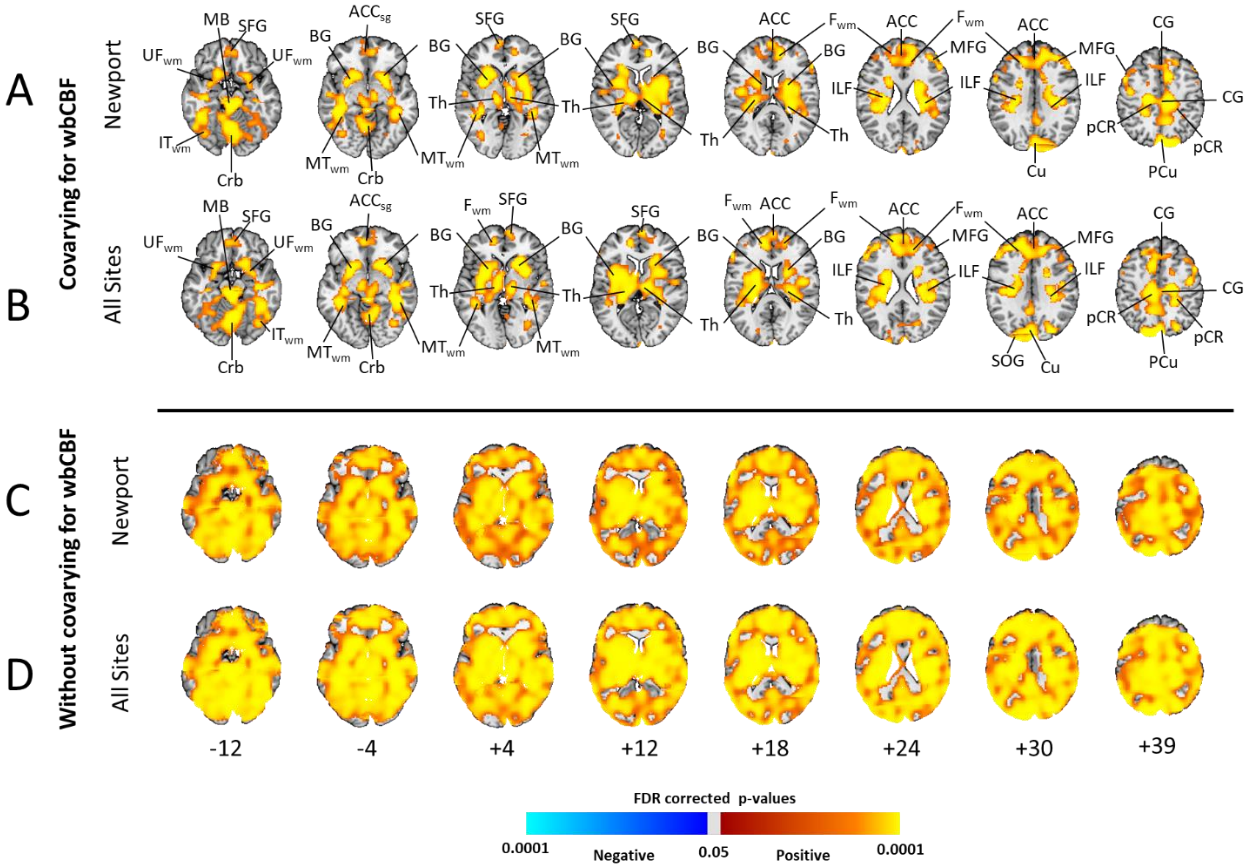

SFigure 1F. Group Differences in Medication-Free Participants. Resting rCBF with and without covarying for wbCBF. At the time of scan, 100 Newport patients and 213 patients across all sites were not taking psychotropic medication. Rows A & B: while covarying for wbCBF. Rows C & D: without covarying for wbCBF. Abbreviations are as in Figure 1 of the main text.

Newport-Only Depressed N=100 (49 males, 51 females, mean age 36.3 years)

Newport-Only Healthy Controls N=78 (33 males, 45 females, mean age 37.9 years)

All Sites Depressed N=213 (99 males, 114 females, mean age 34.8)

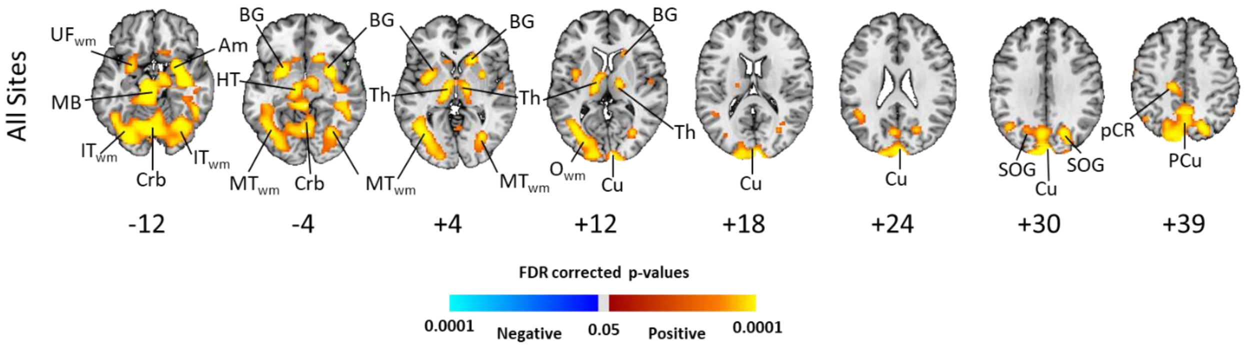

SFigure 1G. Group Differences When Excluding Patients with Comorbid Disorders. Resting rCBF comparing rCBF in 56 depressed patients across all sites with 78 healthy controls from the Newport site while covarying for age, sex, and wbCBF. Abbreviations are as in Figure 1 of the main text.
Newport-Only Healthy Controls N=78 (33 males, 45 females, mean age 37.9 years)
All Sites Depressed Patients excluding patients with co-occurring diagnoses of anxiety disorder, ADHD, OCD, eating disorder, or substance use disorder N=56 (24 males, 32 females, mean age 38.9)

MODIFYING EFFECTS OF AGE ON GROUP DIFFERENCES

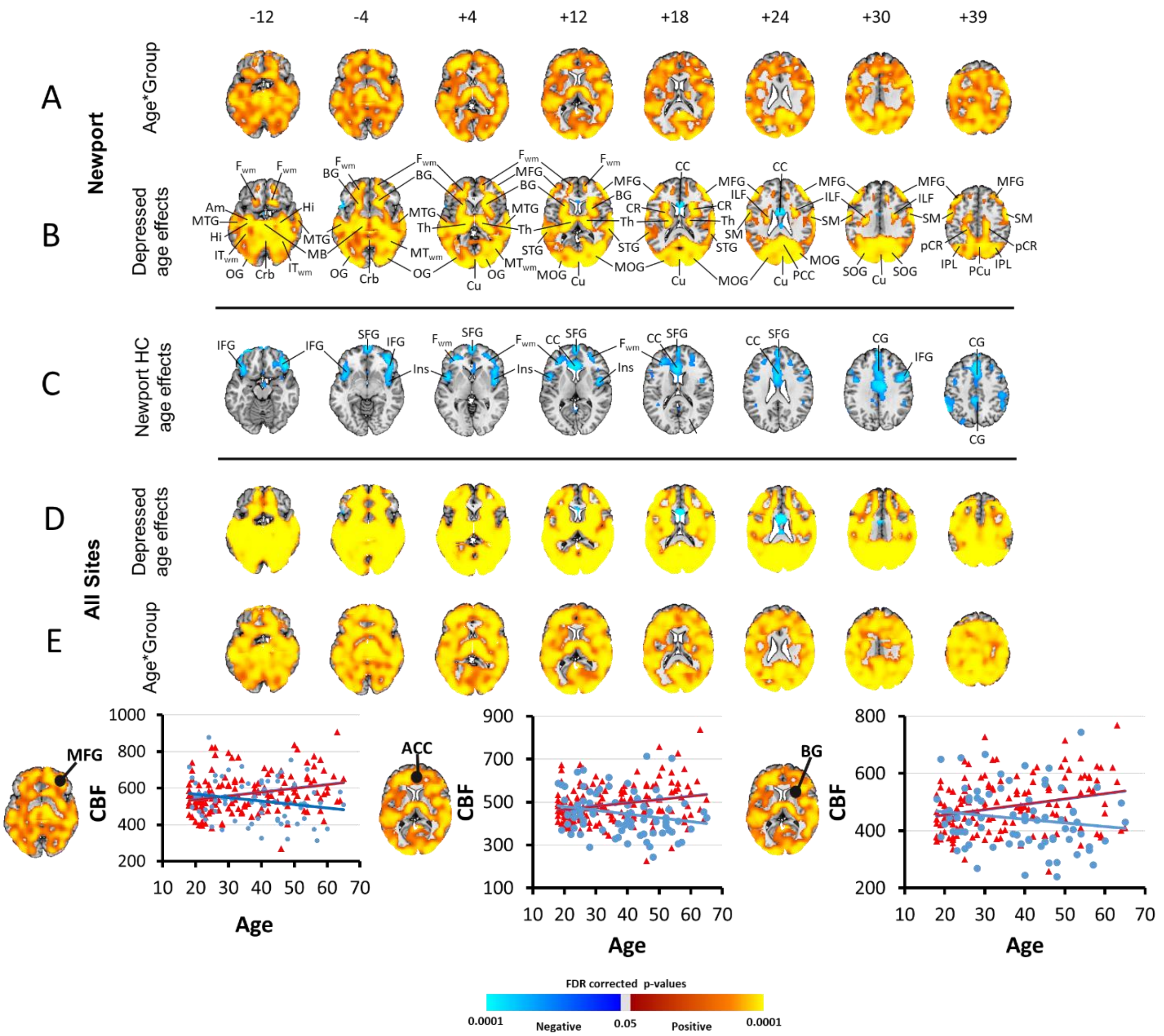

SFigure 2A. Modifying Effects of Age on Resting rCBF Without Covarying for wbCBF

All maps while covary for sex but not for wbCBF. Abbreviations the same as in Figure 1 of the main text.

Row A: Significance map of the age*group interaction in Newport-only participants

Row B: Significance map for associations of age with rCBF in the Newport-only depressed patients

Row C: Significance map for age associations with rCBF in the (Newport-only) healthy controls

Row D: Significance map for associations of age with rCBF in depressed patients across all 8 sites

Row E: Significance map of the age*group interaction in participants across all sites

Bottom: Scatterplots from data at representative points in the brain showing age associations in each group separately.

Newport Only Depressed: N=147 (66 males, 81 females, mean age: 36.1 years)

All 8 Sites Depressed: N=302 (133 Males, 169 Females, Mean Age 35.5 years)

Newport Only Healthy Controls: N=78 (33 males, 45 females, mean age 37.9 years)

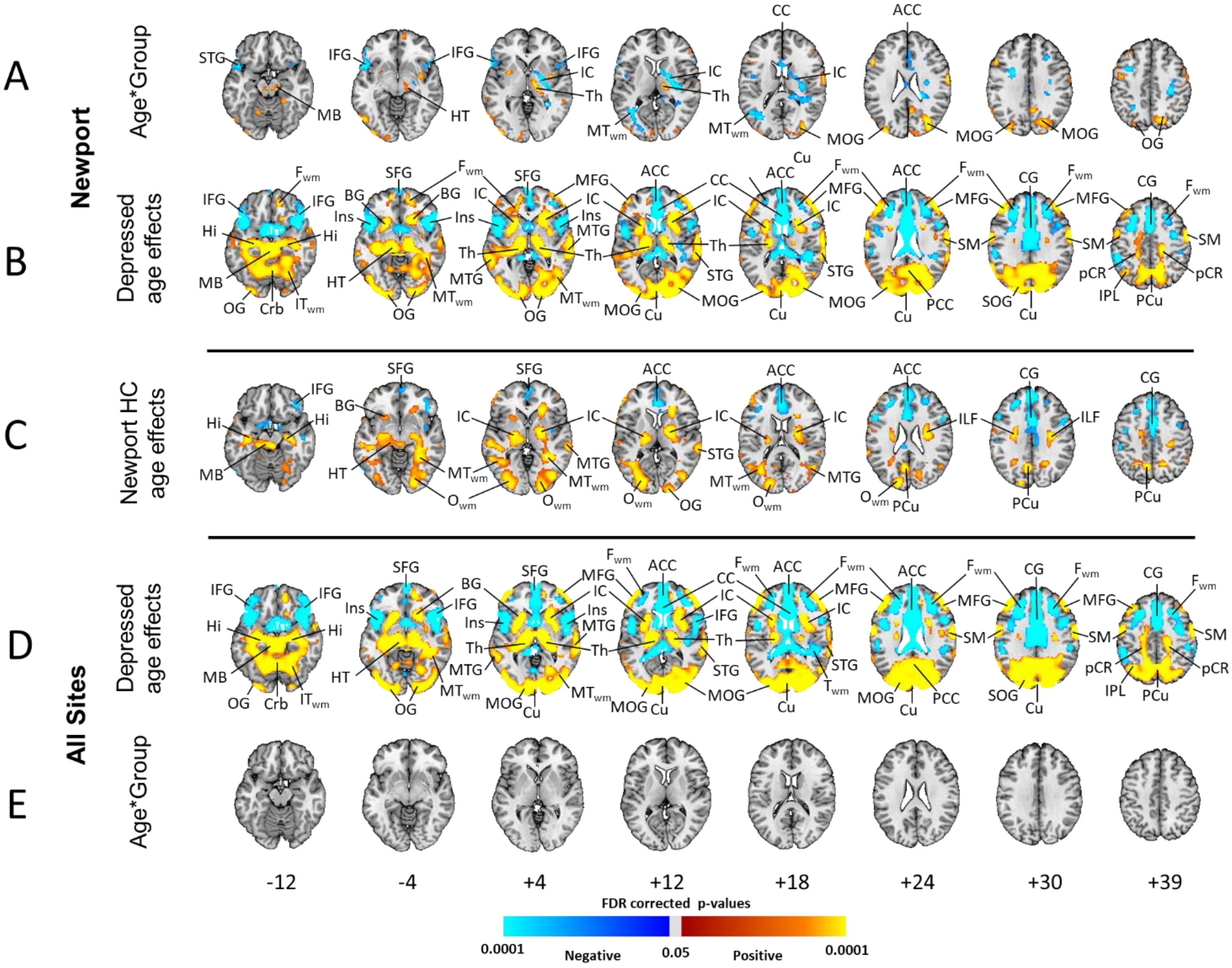

SFigure 2B. Modifying Effects of Age on rCBF During CPT While Covarying for wbCBF.

All maps while covary for sex and wbCBF. Abbreviations the same as in Figure 1 of the main text.
Row A: Significance map of the age*group interaction in Newport-only participants
Row B: Significance map for associations of age with rCBF in the Newport-only depressed patients
Row C: Significance map for age associations with rCBF in the (Newport-only) healthy controls
Row D: Significance map for associations of age with rCBF in depressed patients across all 8 sites
Row E: Significance map of the age*group interaction in participants across all sites
Newport Only Depressed: N=167 (76 males, 91 females, mean age: 36.6 years)
All 8 Sites Depressed: N=336 (147 Males, 189 Females, Mean Age 35.7 years)
Newport Only Healthy Controls: N=103 (43 males, 60 females, mean age: 39.6 years)

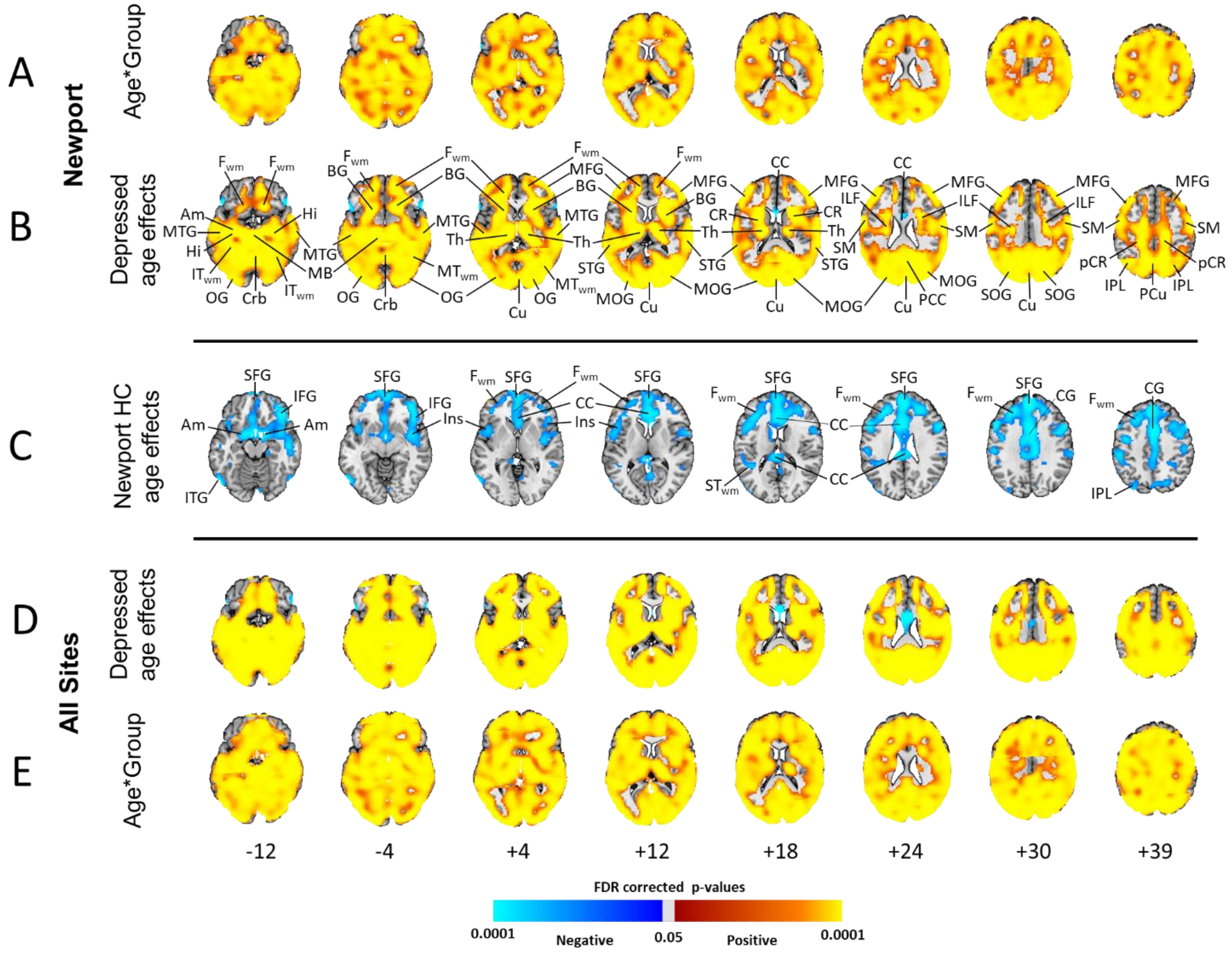

SFigure 2C. Modifying Effects of Age on rCBF during CPT without covarying for wbCBF.

All maps while covary for sex but not for wbCBF. Abbreviations the same as in Figure 1 of the main text.

Row A: Significance map of the age*group interaction in Newport-only participants

Row B: Significance map for associations of age with rCBF in the Newport-only depressed patients

Row C: Significance map for age associations with rCBF in the (Newport-only) healthy controls

Row D: Significance map for associations of age with rCBF in depressed patients across all 8 sites

Row E: Significance map of the age*group interaction in participants across all sites

Newport Only Depressed: N=167 (76 males, 91 females, mean age: 36.6 years)

All 8 Sites Depressed: N=336 (147 Males, 189 Females, Mean Age 35.7 years)

Newport Only Healthy Controls: N=103 (43 males, 60 females, mean age: 39.6 years)

MODIFYING EFFECTS OF SEX ON GROUP DIFFERENCES

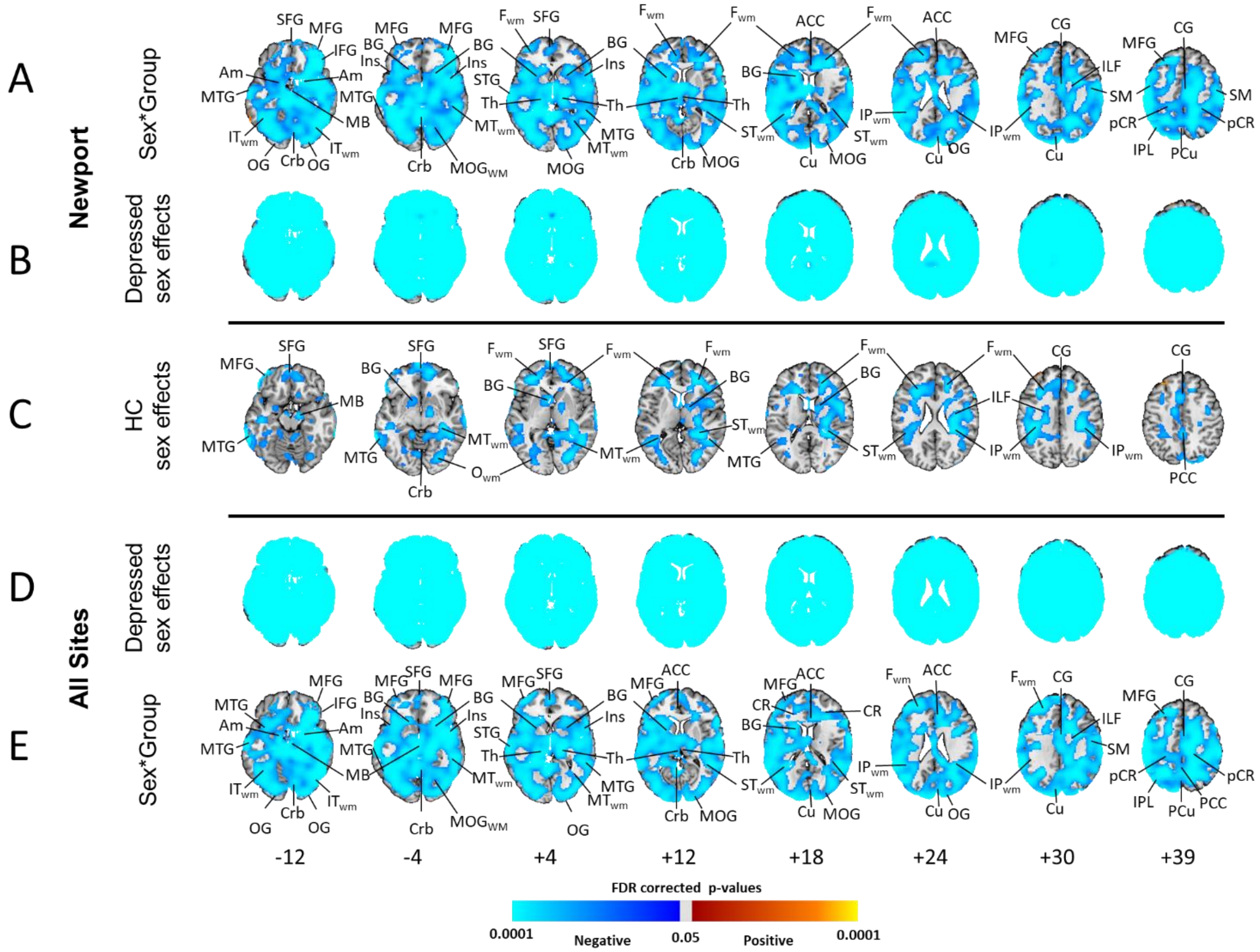

SFigure 3A. Modifying Effects of Sex on Resting rCBF Without Covarying for wbCBF

All maps while covary for age but not for wbCBF. Coding: males=1, females=0. Abbreviations the same as in Figure 1 of the main text.

Row A: Significance map of the sex*group interaction in Newport-only participants

Row B: Significance map for associations of sex with rCBF in the Newport-only depressed patients

Row C: Significance map for sex associations with rCBF in the (Newport-only) healthy controls

Row D: Significance map for associations of sex with rCBF in depressed patients across all 8 sites

Row E: Significance map of the sex*group interaction in participants across all sites

Newport Only Depressed: N=147 (66 males, 81 females, mean age: 36.1 years)

All 8 Sites Depressed: N=302 (133 Males, 169 Females, Mean Age 35.5 years)

Newport Only Healthy Controls: N=78 (33 males, 45 females, mean age 37.9 years)

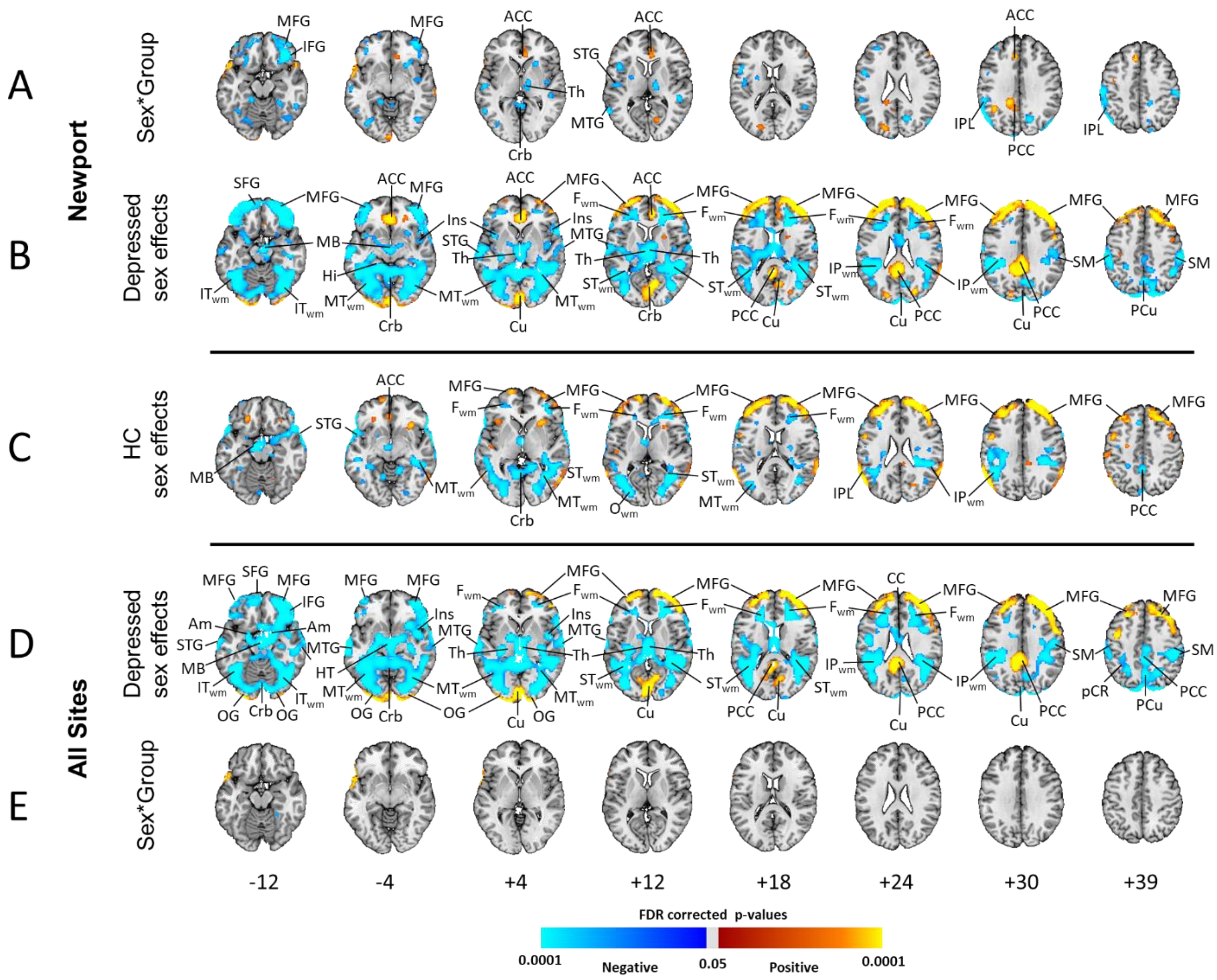

SFigure 3B. Modifying Effects of Sex on rCBF During CPT While Covarying for wbCBF

All maps while covary for age and wbCBF. Abbreviations the same as in Figure 1 of the main text.
Row A: Significance map of the sex*group interaction in Newport-only participants
Row B: Significance map for associations of sex with rCBF in the Newport-only depressed patients
Row C: Significance map for sex associations with rCBF in the (Newport-only) healthy controls
Row D: Significance map for associations of sex with rCBF in depressed patients across all 8 sites
Row E: Significance map of the sex*group interaction in participants across all sites
Newport Only Depressed: N=167 (76 males, 91 females, mean age: 36.6 years)
All 8 Sites Depressed: N=336 (147 Males, 189 Females, Mean Age 35.7 years)
Newport Only Healthy Controls: N=103 (43 males, 60 females, mean age: 39.6 years)

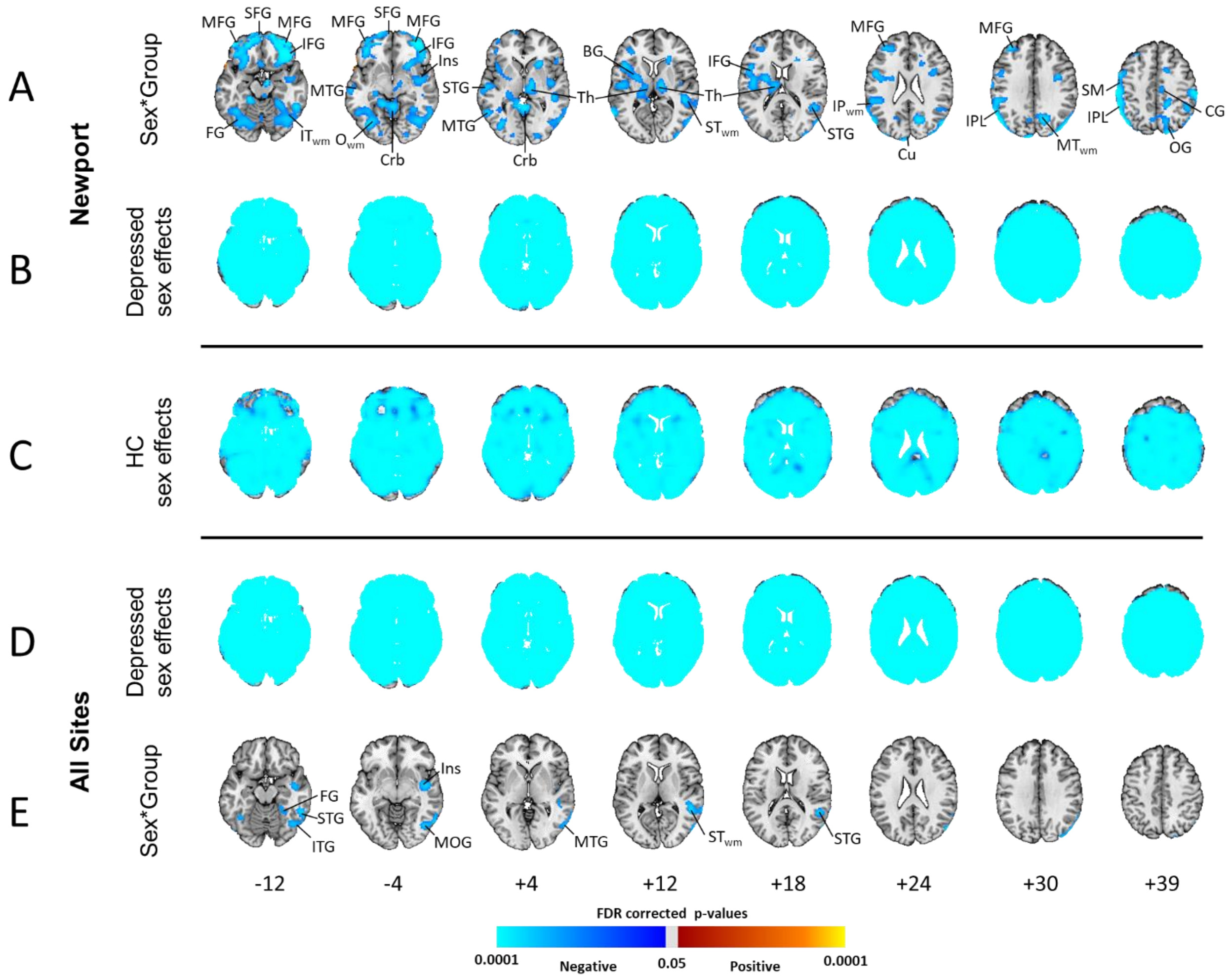

SFigure 3C. Modifying Effects of Sex on rCBF During CPT Without Covarying for wbCBF

All maps while covary for age but not for wbCBF. Abbreviations the same as in Figure 1 of the main text.
Row A: Significance map of the sex*group interaction in Newport-only participants
Row B: Significance map for associations of sex with rCBF in the Newport-only depressed patients
Row C: Significance map for sex associations with rCBF in the (Newport-only) healthy controls
Row D: Significance map for associations of sex with rCBF in depressed patients across all 8 sites
Row E: Significance map of the sex*group interaction in participants across all sites
Newport Only Depressed: N=167 (76 males, 91 females, mean age: 36.6 years)
All 8 Sites Depressed: N=336 (147 Males, 189 Females, Mean Age 35.7 years)
Newport Only Healthy Controls: N=103 (43 males, 60 females, mean age: 39.6 years)

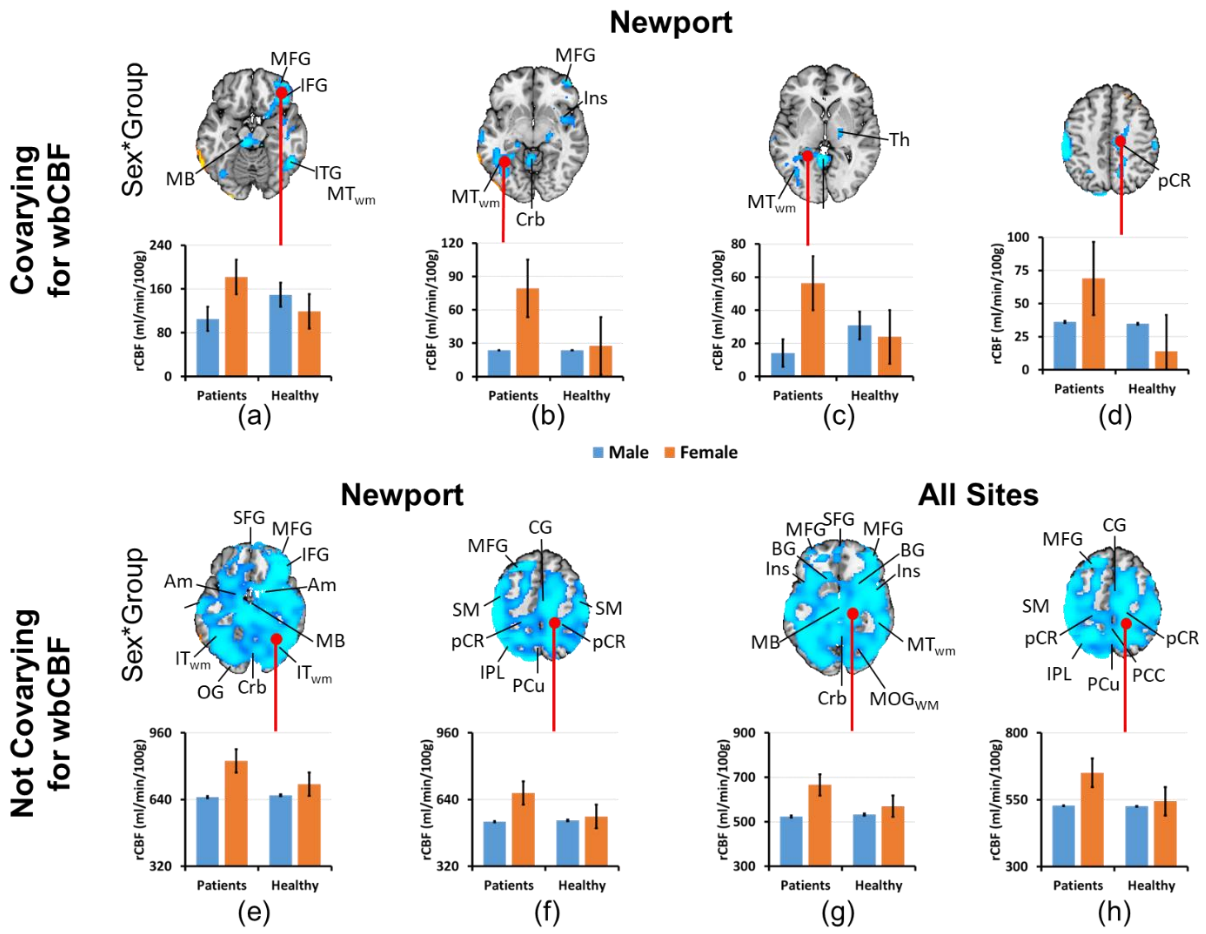

SFigure 3D. Bar Graphs for the Modifying Effects of Sex on Group Differences

Red dots: locations where SPECT data were sampled. Mean values shown above (adjusted for covariates) follow (values are mean (Standard Error, [confidence interval for the mean]):

Plots a-d: rCBF values are adjusted for age and wbCBF.

(a) Patient Males 105.2 (18.99 [67.99 142.46]), Females 181.8 (8.62 [164.92 198.71]);
Healthy Males 149.3 (12.93 [123.95 174.64]), Females 118.9 (10.41 [98.51 139.31])

(b) Patient Males 23.6 (10.04 [3.90 43.24]), Females 79.2 (7.91 [63.71 94.70]);
Healthy Males 23.7 (14.19 [-4.16 51.50]), Females 27.6 (8.71 [10.50 44.65])

(c) Patient Males 14.2 (8.64 [-2.77 31.09]), Females 56.4 (5.77 [45.05 67.66])
Healthy Males 30.8 (9.07 [13.06 48.62]), Females 23.9 (7.8 [8.57 39.15])

(d) Patient Males 36.2 (7.59 [21.28 51.02]), Females 69.0 (6.15 [56.91 81.00])
Healthy Males 34.7 (9.23 [16.58 52.78]), Females 13.9 (7.00 [0.17 27.63])

Plots e-g: rCBF values are adjusted for age but NOT for wbCBF.

(e) Patient Males 651.6 (14.23 [623.75 679.52]), Females 825.6 (16.43 [793.41 857.8])
Healthy Males 660.5 (25.88 [609.76 711.21]), Females 713.8 (23.16 [668.41 759.21])

(f) Patient Males 534.0 (11.35 [511.79 556.3]), Females 671.3 (15.4 [641.09 701.45])
Healthy Males 540.2 (20.78 [499.46 580.93]), Females 558.9 (17.69 [524.21 593.57])

(g) Patient Males 523.3 (8.96 [505.71 540.84]), Females 666.1 (10.25 [645.98 686.14])
Healthy Males 532.5 (21.75 [489.91 575.17]), Females 570.5 (19.9 [531.52 609.53])

(h) Patient Males 528.1 (8.72 [511.05 545.21]), Females 651.3 (9.41 [632.82 669.7])
Healthy Males 525.9 (21.05 [484.65 567.17]), Females 544.4 (17.81 [509.46 579.27])

ASSOCIATIONS WITH SYMPTOM SEVERITY

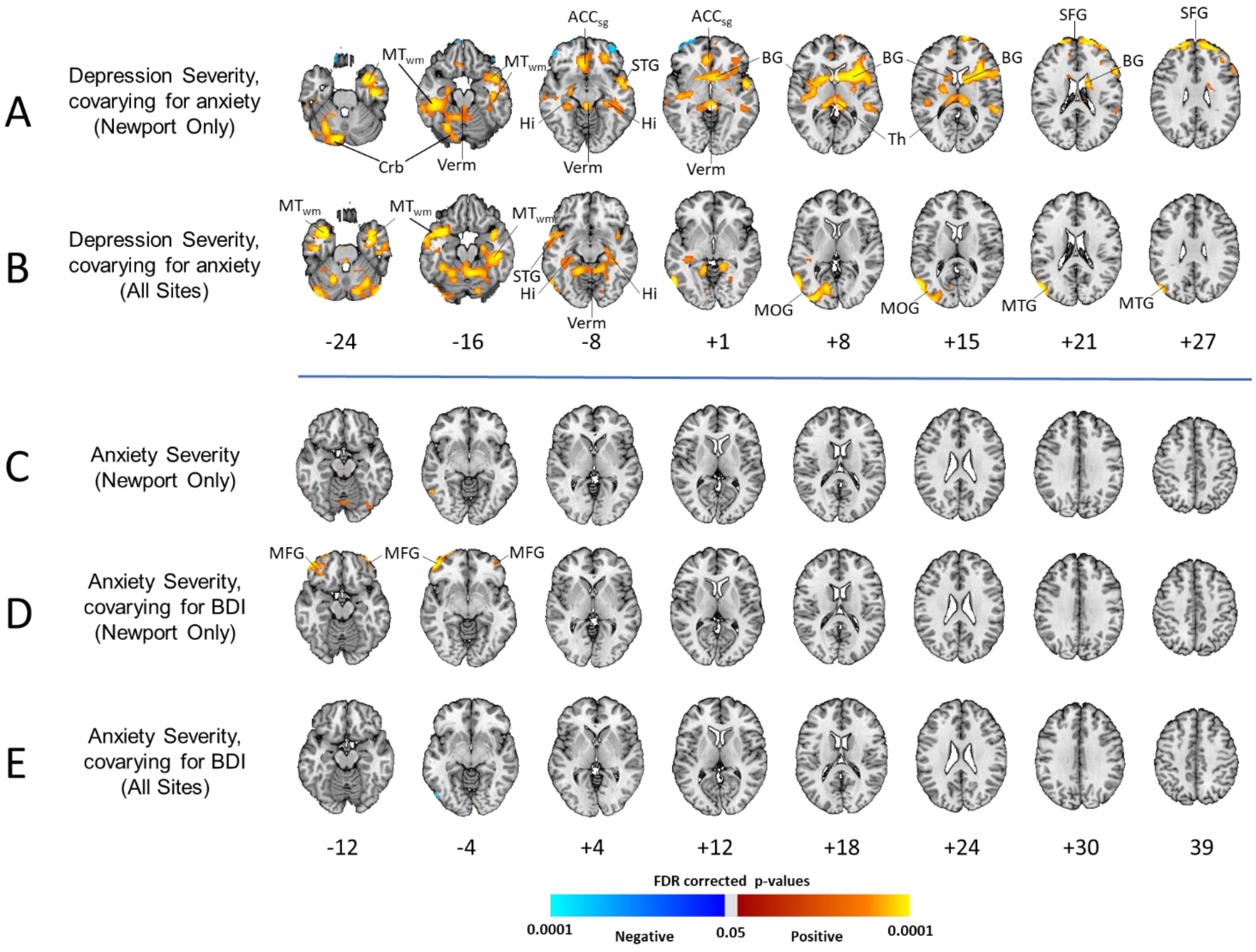

SFigure 4A. Associations of Depression & Anxiety Symptom Severity with Resting rCBF While Covarying for wbCBF

Depression severity was assessed using the Beck Depression Inventory (BD). Anxiety severity was assessed using a DSM-based questionnaire for anxiety symptoms (see SM-Methods). The correlation of BDI and anxiety severity scores across all sites was r=0.53 (p<0.005).

Row A: Association of depression symptom severity with rCBF in Newport-only patients while covarying for age, sex, wbCBF, and anxiety symptom severity. N=110 (44 males, 66 females, mean age 36.6)

Row B: Association of depression symptom severity with rCBF in patients across all 8 sites while covarying for age, sex, wbCBF, and anxiety symptom severity. N=213 (80 males, 133 females, mean age 36.2)

Row C: Association of anxiety symptom severity with rCBF in Newport-only patients while covarying for age, sex, and wbCBF. N=129 (57 males, 72 females, mean age 36.3 years)

Row D: Association of anxiety symptom severity with rCBF in Newport-only patients while covarying for age, sex, wbCBF, and BDI scores. N=110 (44 males, 66 females, mean age 36.6)

Row E: Association of anxiety symptom severity with rCBF in patients across all 8 sites while covarying for age, sex, wbCBF, and BDI scores. N=213 (80 males, 133 females, mean age 36.2)

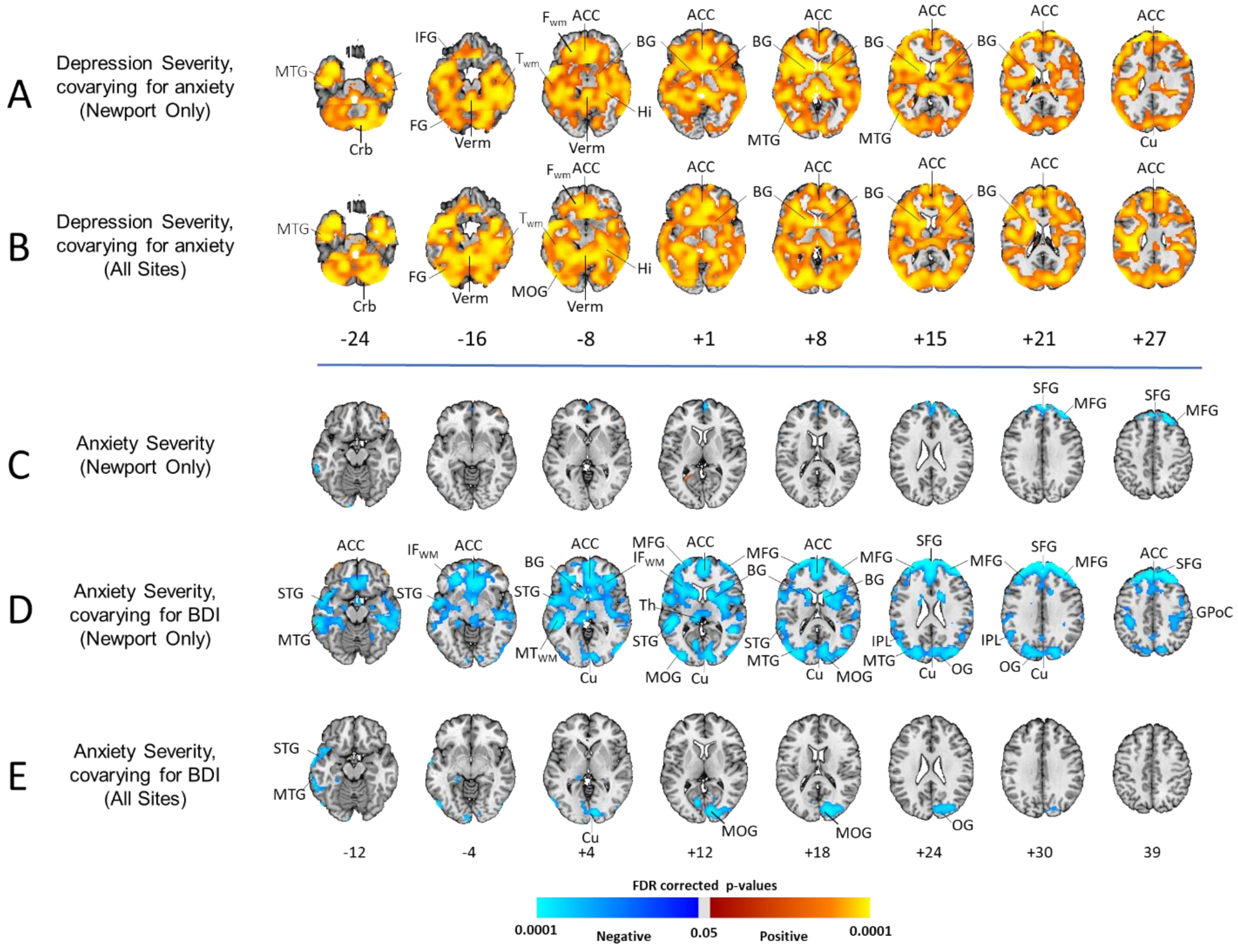

SFigure 4B. Associations of Depression & Anxiety Symptom Severity with Resting rCBF Without Covarying for wbCBF.

Depression severity was assessed using the Beck Depression Inventory (BD). Anxiety severity was assessed using a DSM-based questionnaire for anxiety symptoms (see SM-Methods). The correlation of BDI and anxiety severity scores across all sites was r=0.53 (p<0.005).

Row A: Association of depression symptom severity with rCBF in Newport-only patients while covarying for age, sex, and anxiety symptom severity. N=110 (44 males, 66 females, mean age 36.6)

Row B: Association of depression symptom severity with rCBF in patients across all 8 sites while covarying for age, sex, and anxiety symptom severity. N=213 (80 males, 133 females, mean age 36.2)

Row C: Association of anxiety symptom severity with rCBF in Newport-only patients while covarying for age, and sex. N=129 (57 males, 72 females, mean age 36.3 years)

Row D: Association of anxiety symptom severity with rCBF in Newport-only patients while covarying for age, sex, and BDI scores. N=110 (44 males, 66 females, mean age 36.6)

Row E: Association of anxiety symptom severity with rCBF in patients across all 8 sites while covarying for age, sex, and BDI scores. N=213 (80 males, 133 females, mean age 36.2)

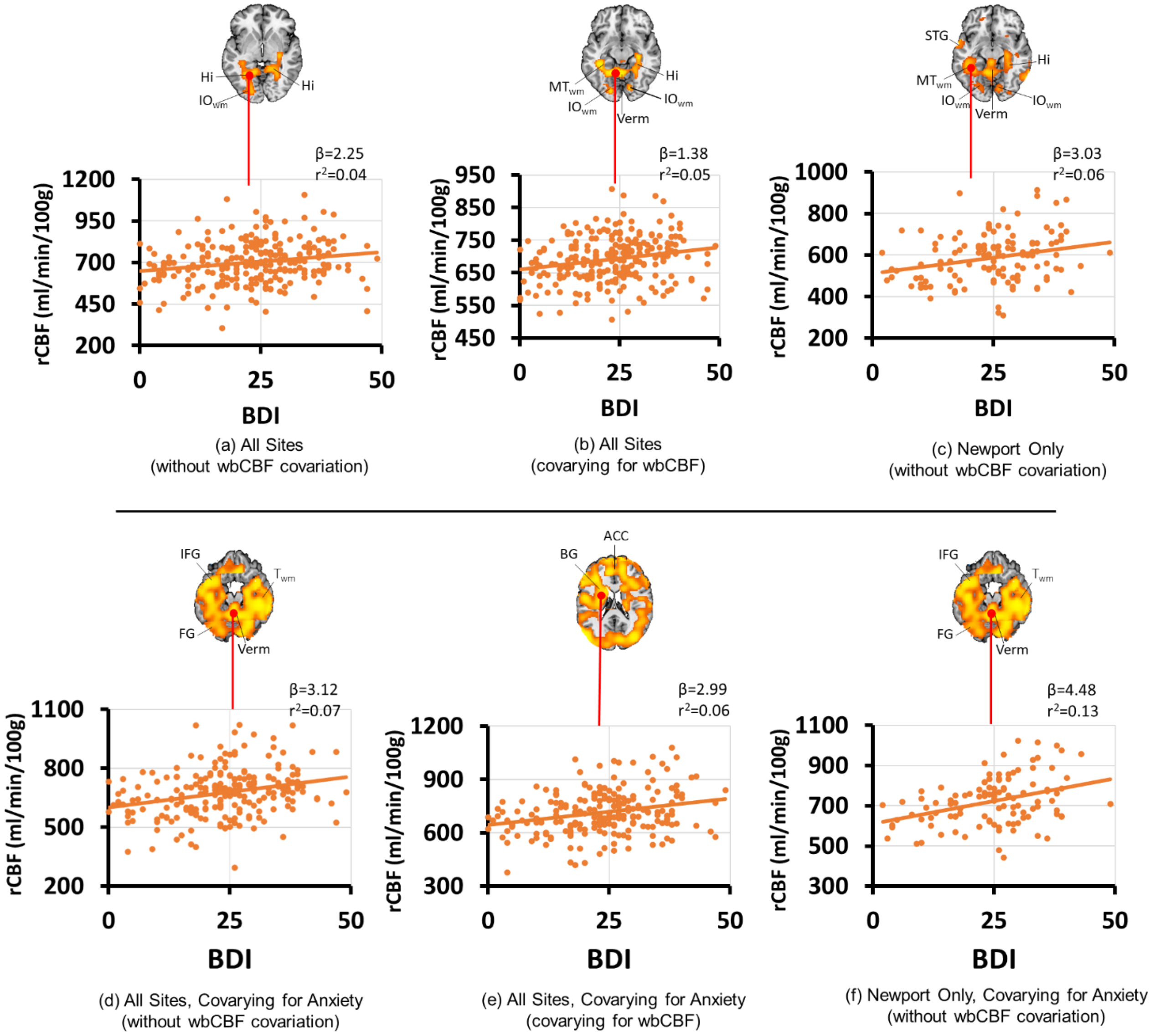

SFigure 4C. Scatterplots for Depression Severity Associations.
The red dot indicates where rCBF data were sampled. All regression models covaried for age and sex. The statistical maps a-c are slices taken from Figure 4 of the main text, and maps d-f are slices taken from SFigure 4B.

MEDICATION EFFECTS

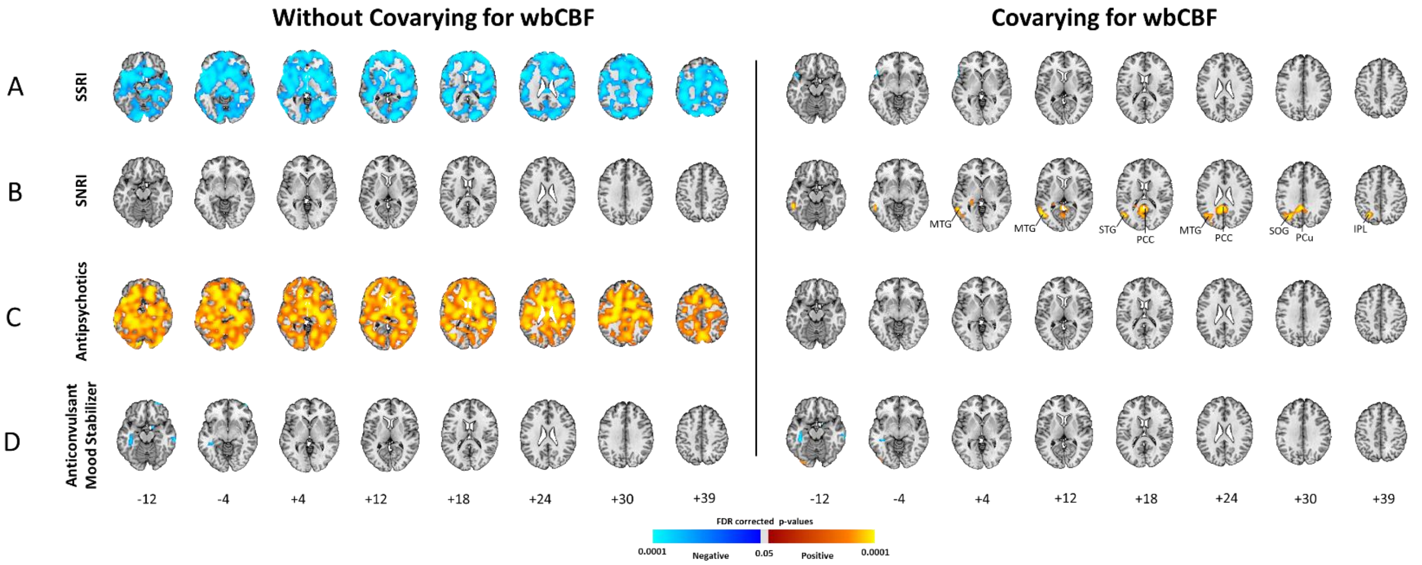

SFig.5. Psychotropic Medication Effects on rCBF at Rest With and Without Covarying for wbCBF.
Row A: SRIs (Newport N=30, all sites N=47)
Row B: SNRIs (Newport N=10, all sites N=19)
Row C: Antipsychotics (Newport N=4, all sites N=18)
Row D: Anticonvulsant mood stabilizers (Newport N=5, all sites N=10)
Lithium (Newport N=1, all sites N=3) – null findings (not shown)
All analyses covary for age and sex but not for wbCBF. Analyses including subjects from all sites control for site. Warm colors indicate significantly increased rCBF and cool colors indicate significantly less rCBF associated with psychotropic use in depressed patients. The Z-values represent slice levels (in millimeters) in the Talairach coordinate system.
